# Operando Scanning Electron Microscopy Study of Support Interactions and Mechanisms of Salt-Assisted WS_2_ Growth

**DOI:** 10.1021/acs.chemmater.4c02603

**Published:** 2025-01-30

**Authors:** Jinfeng Yang, Ye Fan, Ryo Mizuta, Max Rimmer, Jack Donoghue, Shaoliang Guan, Sarah J. Haigh, Stephan Hofmann

**Affiliations:** † Department of Engineering, University of Cambridge, Cambridge CB3 0FA, U.K.; ‡ Department of Materials, University of Manchester, Manchester M13 9PL, U.K.; § Maxwell Centre, Cavendish Laboratory, University of Cambridge, Cambridge CB3 0HE, U.K.

## Abstract

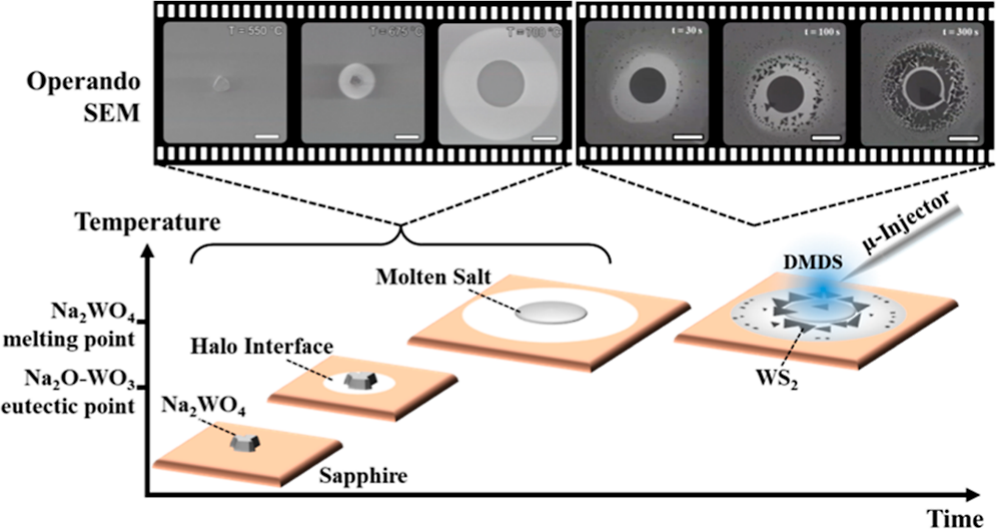

Salt enhanced chemical vapor deposition of WS_2_ and related 2D materials is widespread, and while many mechanisms including vapor–liquid–solid (VLS) mediated growth have been suggested, gaining a more detailed understanding remains challenging. We employ operando scanning electron microscopy to resolve the entire process of salt-assisted CVD of WS_2_, focusing on a model system of individual, small (<100 μm), sapphire supported sodium tungstate (Na_2_WO_4_) salt particles. We reveal support interactions that lead a salt particle to develop a lateral halo interface, driven by surface eutectic melting above 630 °C. This halo dictates the salt wetting as well as Na and W transport, and thus upon gaseous sulfur precursor exposure dominates the spatiotemporal WS_2_ nucleation and mono- and multilayer domain expansion kinetics, all of which we can directly track by secondary electron (SE) contrast with a conventional In-Lens SE detector. Unlike for a conventional VLS mechanism, large (>20 μm) monolayer WS_2_ formation does not involve the salt droplet directly attached to the growth facets, rather the salt droplet drives WS_2_ layer growth in the contiguous halo interface region with a continuous supply of W. We compare this to SiO_2_ and NaOH treated sapphire where corrosive surface roughening dictates the salt wetting, and critically discuss our findings in the context of the connected wider literature.

## Introduction

The technological potential of atomically thin device materials, such as the family of transition metal dichalcogenides (TMDs), hinges on the viability of heterogeneous integration with existing materials and process flows, and as echoed across industrial roadmaps, the bottleneck is nanomanufacturing technology to enable scalability at sufficiently high level of structural control.^1−3^ Chemical vapor deposition (CVD) approaches are most promising in this context, reflected by a large body of literature of powder-based CVD, metal organic (MO)CVD and atomic layer deposition (ALD) of TMD mono/few-layer films.^4−11^ The use of salts as CVD process promotors is becoming increasingly widespread for these approaches. This ranges from the use of alkali metal halide salts, e.g. NaCl, to increase metal powder precursor volatilisation,^12−14^ their use in substrate pretreatment to achieve significantly larger crystal domain sizes,^4,15−17^ higher growth rates and more benign/lower temperature process conditions.^18,19^ Salts such as Na molybdates and tungstates can serve as metal source allowing more effective feeding, area-selective growth and doping.^20−28^ As a trade-off, however, there remain many open questions not only regarding the impurity and defect levels of the as-grown TMDs,^17,29,30^ but for transfer-free, digital processing also regarding the level of substrate interaction and possible hot-salt driven corrosion, particularly for commonly used SiO_2_ and sapphire support.^31,32^

Many underpinning mechanisms have been proposed specifically for the role of salts on the surface of the substrate, ranging from effective barrier lowering at 1D reaction fronts,^33−38^ the formation of surface/eutectic intermediates promoting precursor feeding to the growing facets,^39−41^ to vapor–liquid–solid (VLS) type growth modes, where a liquid droplet stably wets the growth front(s) and locally mediates dissociation and/or incorporation of species.^42−49^ Progressing such understanding, however, remains challenging as conventional experimentation only allows post-mortem characterization, i.e. there is a critical lack of data on what actually happens during the process. Complete or ab initio modeling^50^ of such complex, multistep and multiscale growth is currently not possible. Operando experimentation has been key to the discovery of growth mechanisms,^51−54^ but accessing the vast CVD parameter space across nm to mm multi size scales remains difficult.

Here, we use operando scanning electron microscopy (SEM) to directly interrogate the mechanisms of surface-bound salt-assisted WS_2_ layer CVD. We focus on a model system of individual, small (<100 μm) tungstate salt (Na_2_WO_4_) particles on c-plane sapphire support. Using secondary electron (SE) contrast with a conventional In-Lens SE detector, we can directly track the support interactions during vacuum annealing, the specifics of which dictate the salt wetting as well as Na and W transport. Upon gaseous sulfur precursor exposure these interactions also determine the spatiotemporal WS_2_ nucleation and mono- and multilayer domain expansion kinetics. Unlike to a conventional VLS mechanism, large (>20 μm) monolayer WS_2_ formation does not involve the salt droplet directly attached to the growth facets, rather the salt droplet drives WS_2_ layer growth in a contiguous halo interface region with a continuous supply of W. We present operando SEM video data sets with systematic pre/postgrowth sample characterization by cross-sectional transmission electron microscopy (TEM), energy dispersive X-ray spectroscopy (EDS), X-ray photoelectron spectroscopy (XPS), atomic force microscopy (AFM), optical microscopy and Raman and photoluminescence (PL) spectroscopy. We suggest that the support interactions and lateral halo interface formation are driven by surface eutectic melting above 630 °C. We compare the support interactions on sapphire to those on SiO_2_ and NaOH treated sapphire where corrosive surface roughening dictates the salt wetting. Our results highlight the critical role of salt–support interactions in terms of dictating growth mechanisms and for future heterogeneous material and process integration.

## Results

**Figure 1:**
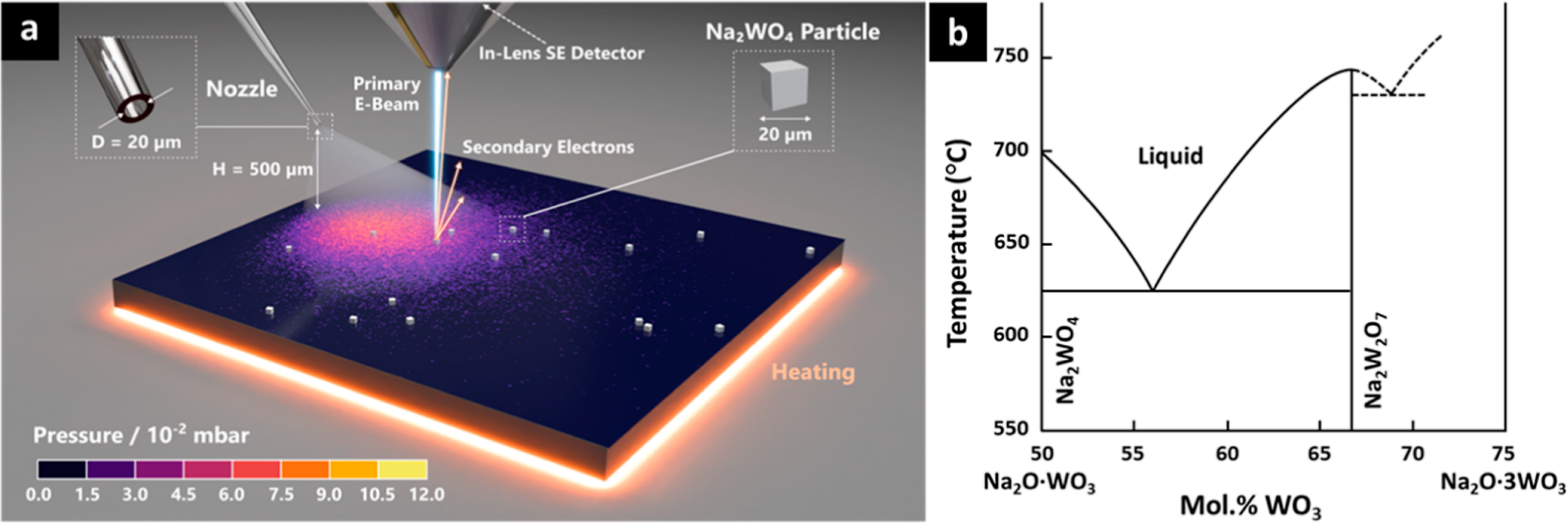
(a) Schematic of the operando SEM setup and model system of sapphire supported Na_2_WO_4_ particles that are heated and then exposed to DMDS gas. (b) Phase diagram of the Na_2_O-WO_3_ system replotted from literature.^56,57^

Figure 1 schematically outlines the model system and operando SEM setup. We use Na_2_WO_4_ salt crystals, well dispersed on c-plane sapphire or SiO_2_/Si wafer supports (see Experimental Section). These substrates are commonly used across the literature, and sapphire is among the most inert substrate options. The salt is the sole supply of W for the WS_2_ synthesis, and has low vapor pressure at the conditions used,^55^ i.e. will promote localized, mainly surface-bound process reactions. A custom-made quartz gas microinjector (μ-I, see Figure S1a,b) is used to supply dimethyl disulfide (DMDS) as the S precursor. Test-particle Monte Carlo (TPMC) simulations show that a typical μ-I opening diameter (D; Figure 1a) of approximately 20 μm and height (H; Figure 1a) of approximately 500 μm above the sample gives a localized high-pressure region of around 0.1 mbar over an estimated 500 μm diameter area of interest on the substrate (see Figure S1c). This allows us to efficiently interrogate the process and parameter space by operando SEM using a high-resolution In-Lens SE detector (see Experimental Section). SEM imaging was preferentially performed at above 500 °C which also mitigates sample charging. The bulk phase diagram of the Na_2_O-WO_3_ system (Figure 1b) indicates that the Na_2_WO_4_ salt crystals are expected to melt around 700 °C, with a eutectic lowering that temperature by approximately 70 °C toward higher WO_3_ concentration (∼56 mol %). The initial process flow thus chosen was to stepwise heat in vacuum (base pressure ∼10^–6^ mbar) up to salt liquefaction (Stage 1) and then expose to DMDS (Stage 2) to form WS_2_ (see Figure S2).

**Figure 2:**
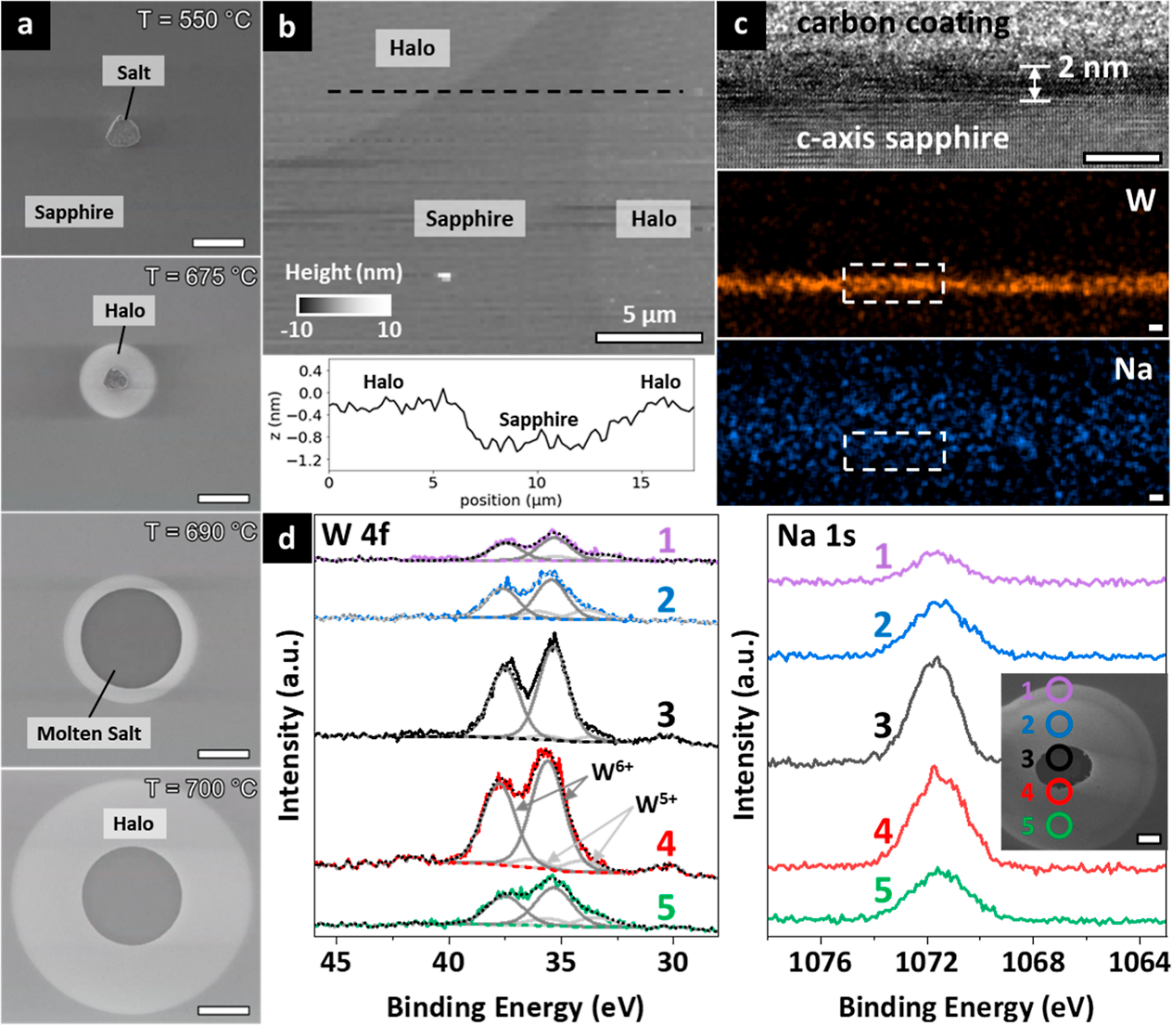
(a) SEM image sequence of stage 1 annealing and melting of a sapphire-supported Na_2_WO_4_ particle. Scale bars = 20 μm. (b) Postannealing AFM map of two adjacent halos (max. *T* = 700 °C, anneal time 1.5 h), measured right after cooling. The dashed line in the map indicates location of the shown line scan. (c) Postannealing cross-sectional TEM and STEM-EDS maps of W and Na of the halo interface (max. *T* = 720 °C, anneal time = 10 min). The white dashed boxes in the STEM-EDS maps correspond to the area of the TEM image. Scale bars = 5 nm. (d) W 4f and Na 1s core level XPS spectra mapped across the droplet and the halo interface (max. *T* = 720 °C, anneal time = 10 min). The positions of points 1–5 are indicated in the inset (scale bar = 20 μm).

Figure 2a shows a representative plan-view operando SEM image sequence (see Video S1 for full SEM video) of stage 1 for a sapphire supported Na_2_WO_4_ salt crystal. The SE contrast that is seen at temperature is distinct to postcooling and postair-exposure characterization, as discussed below. At approximately 630 °C a well-defined brighter concentric ring starts to form around the salt crystal. This appears insensitive to the detailed salt particle shape and sapphire surface crystallography. This halo region expands with time and temperature. At any stage we find the halo strictly circular and with clearly discernible SE contrast for all salt crystals tracked on planar sapphire (>150 in total), across different vacuum annealing conditions and temperature ramps. For temperatures below ∼690 °C the central salt particle remains solid, and while the halo emanates from the salt, the top of the salt crystal initially shows little morphological change. Also, SE contrast variations in the form of concentric rings can be seen within the halo at ∼670–690 °C (see Video S1). Upon further increasing the temperature (*T*) to ≥ ∼ 690 °C full salt particle liquefaction can be observed. The lateral footprint of the salt thereby rapidly increases, and the salt droplet adopts a circular shape of homogeneous SE contrast. Given the comparatively small dimensions of the original solid salt particle, and the fact that the density of the liquid salt does not vary significantly from its solid form,^58^ this implies that the salt droplet adopts a low wetting angle (consistent with postprocess TEM analysis, Figures S5, S8). The salt droplet almost fully wets the existing halo and remains central with respect to the still visible halo. After this melting, the halo continues to concentrically expand while the central liquid droplet does not further laterally expand. For increasing halo extend some shrinking of the droplet can be observed (see Figure S3), i.e. a minor contraction of the lateral wetting footprint. This is consistent with the low vapor pressure of the salt and with continuous transport of material away from the salt into the halo. Blind runs, i.e. without or with significantly lower electron beam exposure also showed the halo around each individual droplet (see Figure S4a), demonstrating that the key features revealed are not electron beam-driven. This is further supported by experiments in a conventional cold-wall low pressure CVD reactor that show similar postannealing characterization results (Figure S4b). We note that the halo is around 30 μm wide for given conditions regardless of droplet size, indicating that the width of the halo is independent of droplet volume/diameter.

We use AFM, cross-sectional TEM, EDS and XPS to characterize the structure and chemical nature of the halo ex-situ. Figure 2b shows an AFM analysis of the halo region after <1 h air exposure, which shows that the surface is relatively flat (root-mean-square roughness <0.5 nm), as expected for these substrates, but that the halo edge/region (∼30 μm to the droplet) is raised by approximately 1 nm. Figure 2c shows cross-sectional TEM analysis of the halo region approximately ∼6 μm away from the outer droplet edge (see Figure S5). For this analysis, the total sample air exposure was again <1 h (see Experimental Section). A ∼2 nm thick layer is resolved above the sapphire support. The corresponding STEM EDS analysis (Figure 2c) confirms the presence of W and Na in this halo surface layer. The Na signal intensity is comparatively lower and Na appears more widely distributed. The Na signal primarily extends into the carbon coating rather than the sapphire substrate, suggesting that this is due to mobility of the Na atoms under the electron beam, i.e. it is distributed by the EDS measurement itself due to its low weight. Figure 2d shows XPS analysis across a representative droplet and its halo, specifically the W 4f and Na 1s core level signatures. The Na 1s binding energies (BEs) are centered around 1071.8 eV. The W 4f signatures can be fitted with two spin–orbit doublets, with the respective W 4f_7/2_ and W 4f_5/2_ BEs being separated by ∼2.2 eV. The BEs for the 4f_7/2_ peak for the assigned W^6+^ oxidation state are centered around ∼35.4 eV. These BEs are consistent with literature values for Na_2_WO_4_.^59,60^ As the interpretation of BEs for salt on insulator samples is challenging, including possible differential charging effects, we focus the line scan analysis on relative fitted peak area fractions of W^6+^ and a lower oxidation state which we assign to W^5+^. We observe a higher fraction of lower W-oxidation toward the outer halo rim compared to the central salt particle. XPS survey scans show no other elements than W, Na, O, Al in these regions, i.e. we can exclude the presence of significant impurity levels (see Figure S6). These findings are also consistent with SEM based EDS characterization (see Experimental Section), which further confirm the presence of Na across the halo region (Figure S7). We further analyzed the area directly underneath the salt droplet by cross-sectional TEM and STEM-mode EDS (Figure S8). We observe a piece of Al_2_O_3_ crystal removed from the sapphire surface, indicative of potential corrosive hot salt effects enhanced possibly by a mismatch between thermal expansion coefficients of sapphire and sodium tungstate.

For postprocess characterization of stage 1, we find that the halo region shows significant changes after prolonged air exposure. After 3 weeks of ambient air exposure AFM shows discrete particles in the halo region with 10 s of nm dimensions (Figure S9). Figure S10a–c show SEM images of a salt droplet with its halo after melting, during cool down (both done by operando SEM) and after air exposure for 5 days at room temperature (postprocess SEM), respectively. For the latter we consistently observe small nanoparticles particularly across the outer rim of the halo region, which based on EDS analysis (Figures S10d–h) contain Na and W. We note that the EDS C map (Figure S10f) also consistently follows this roughening, reflecting selective adsorption of atmospheric carbon species. Figure S11 shows a salt particle annealed only to max. 640 °C for 10 min and then cooled. A halo was formed but the salt particle remained solid, as expected. After 5 days of air exposure we find again nanoparticles, but this time also outside the original halo. We can thus clearly link the roughening and particle formation to the presence of Na. As this only occurs after prolonged air exposure, we propose this reflects a Na reaction with moisture and CO_2_ in air to form e.g. NaOH and Na_2_CO_3_.^61^ Post annealing Raman analysis of a resolidified salt particle (Figure S12; annealed to 700 °C, 3 h) finds a signature consistent with a Na_2_W_2_O_7_ composition, indicating that the Na_2_WO_4_ can undergo compositional changes during stage 1.

**Figure 3:**
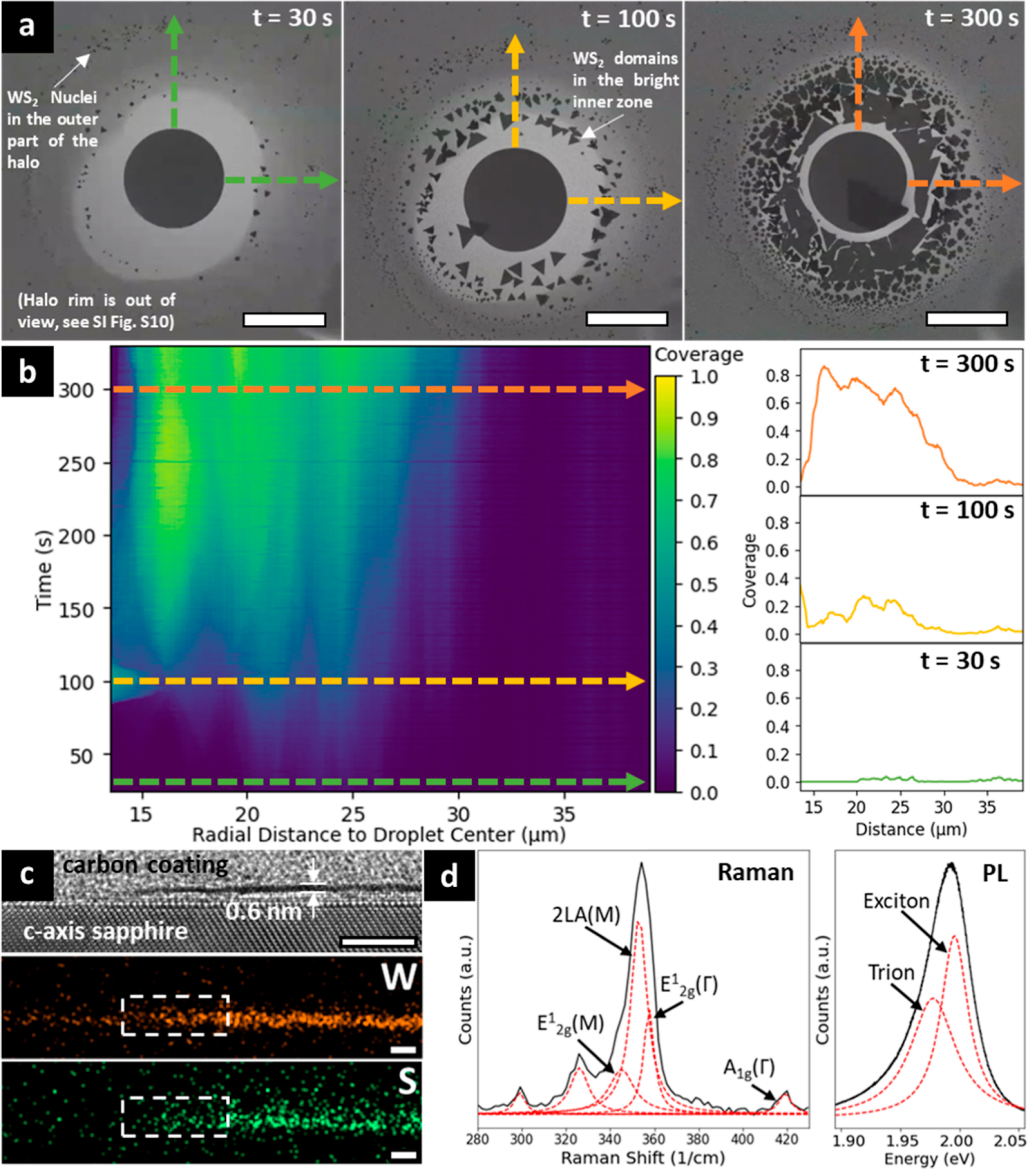
(a) Representative SE image sequence of stage 2, showing WS_2_ nucleation and growth at exposure *t* = 30 s, *t* = 100 s and *t* = 300 s, at 700 °C under 0.1 mbar DMDS. Scale bar = 20 μm. (b) Heat map of WS_2_ coverage at different radial distances to the droplet center versus time, corresponding to the growth event in (a), with the line profiles showing the WS_2_ coverage at different radial distances at *t* = 30 s, *t* = 100 s and *t* = 300 s. (c) Postgrowth cross-sectional TEM of monolayer WS_2_ grown in the halo region at 700 °C under 0.1 mbar DMDS exposure, with corresponding W and S STEM-EDS maps. The area marked by the white boxes in the EDS maps corresponds to the area of the TEM image. Scale bar = 5 nm (d) Raman and PL spectra of as-grown WS_2_ monolayer marked by the yellow box in Figure S16.

Figure 3a shows a representative plan-view SE image sequence of stage 2 (example 1, corresponding to Video S2), when an individual molten salt droplet supported on sapphire and held at 700 °C is exposed to DMDS via the μ-I. We further imaged in excess of 150 salt particles, with Figure S13 and Videos S3, S4 and S5 showing further data sets (examples 2–4) where for instance the μ-I height (H, Figure 1a) was varied, corresponding to different local DMDS exposure pressures (see Figure S1c,d). Figure 3c,d show postprocess cross-sectional TEM, Raman and PL analysis of the halo region around a salt particle. As discussed in further detail below, we can clearly show that the operando SE contrast seen upon DMDS exposure corresponds to WS_2_ nucleation and growth, initially constrained to monolayer thickness. A common finding for all videos is that there is no immediate WS_2_ nucleation directly on the liquid salt droplet. Rather WS_2_ nucleation occurs in the halo region formed at stage 1. There is also no nucleation outside the halo region and the halo does not expand throughout stage 2, as highlighted in examples 3 and 4 (see Figure S13 and Videos S4 and S5) and Figure S14, the postgrowth optical and SE images of example 1.

At the very beginning of stage 2, a high WS_2_ nucleation density can be seen in the outer part of the halo (see also example 3 and 4 in Figure S13, and the corresponding Videos S4 and S5), from the outer rim of the halo to around 15 μm radial distance to the edge of the droplets. This part of the halo loses its bright SEM appearance immediately upon the DMDS exposure, with a bright inner zone (i.e., area of higher SE yield, see Figures 3a and S13) that is not strictly circular anymore maintained only close to the salt droplet. Then in this bright inner zone, WS_2_ nucleation gradually occurs concentrically inward (see all four growth examples, Supporting Information). This is reflected by the heat map in Figure 3b, which shows the WS_2_ coverage as a function of time and position in example 1 (corresponding to Figure 3a and Video S2). The WS_2_ coverage at a certain time and radial distance to the center of the droplet is thereby defined by the percentage of the pixels showing the SE contrast of WS_2_ with respect to all pixels within that radial distance. While the WS_2_ nuclei in the outer part of the halo do not further expand, in the inner zone we observe continued in-plane WS_2_ domain growth. The heat map (Figure 3b) highlights that the average WS_2_ growth rate is increasing with decreasing distance from the droplet. Large (>20 μm) WS_2_ domains thus only form close to the salt droplet. At the early growth phase many of these WS_2_ domains exhibit equilateral triangle shape. This is consistent with the symmetry of the WS_2_ 1H crystal structure and kinetic selection due to one slowest growing, thus dominating 1D reaction facet. We can extrapolate typical max. lateral WS_2_ growth rates at 700 °C under 0.1 mbar DMDS of around 75 nm/s in the inner halo region. The growth rate decreases with decreasing DMDS pressure and is approximately 15 nm/s at 700 °C under 0.02 mbar DMDS (see Figure S13 and Video S5). The WS_2_ domains are typically not epitaxially aligned.

Following the WS_2_ growth in the halo region, for some droplets we also observe a scenario where a WS_2_ domain nucleated in the inner halo expands and gets in direct contact with the salt droplet. Upon such contact, a rapid growth of multilayer WS_2_ is triggered on the liquid salt surface. This leads to distortions of the salt droplet wetting due to pinning at the contact/growth site, and in some cases to a local retreat and temporal distortion of the spherical droplet wetting footprint (see e.g. Video S2). The growing WS_2_ domain shape on the liquid is roughly triangular, consistent with the low wetting angle of the droplet. The WS_2_ growth rate directly on the droplet surface is significantly higher (>1 μm/s). Further, these WS_2_ domains on the droplet either disappear or are torn apart and separate from their part outside the droplet (see e.g. Video S2). Eventually, a region devoid of WS_2_ forms around the salt droplet and its spherical wetting footprint is restored.

Figure 3c shows postgrowth cross-sectional TEM analysis of WS_2_ grown within the inner halo region (see also Figure S15). The layer is ∼0.6 nm thick, consistent with monolayer WS_2_.^62^ The layer separation to the sapphire surface is ∼0.6 nm, indicating that the ∼1–2 nm thick halo surface layer found at stage 1 (Figure 2c) has reacted away in this region. STEM-mode EDS mapping (Figure 3c) consistently shows W and S for the WS_2_ monolayer, but also indicates some presence of W on the sapphire surface not covered by WS_2_. Figure 3d shows the Raman and PL spectrum of an as-grown WS_2_ domain in a halo region which is marked by a yellow box in the corresponding optical image in Figure S16. The Raman peaks (Figure 3d) can be assigned to the E^1^_2g_ (M) mode at 348.4 cm^–1^, 2LA (M) peak at 353.4 cm^–1^, E^1^_2g_ (Γ) at 357.7 cm^–1^, A_1g_ (Γ) at 419.2 cm^–1^, and 2LA(M)-2E^2^_2g_(Γ) and 2LA(M)-E^2^_2g_(Γ) peaks at 298.6 and 325.7 cm^–1^, respectively. These peaks, the 61.5 cm^–1^ separation of the E^1^_2g_(Γ) and A_1g_(Γ) peaks and the larger intensity of E^1^_2g_(Γ) compared to A_1g_(Γ) are all signatures reported for monolayer WS_2_.^63−65^ The PL spectra (Figure 3d) consistently show characteristic exciton and trion peaks at 1.996 and 1.978 eV, respectively. An important observation for all the growth around droplets is that with increasing exposure time, some monolayer WS_2_ domains in the inner halo region shrink in size, with the then-exposed area being covered by domains showing darker SE contrast. The WS_2_ domains directly on the droplet also show such darker contrast. Consistent with SE contrast studies for 2D materials,^66,67^ this reflects the growth of additional WS_2_ layers. Optical microscopy and PL mapping (Figure S16) further support the growth of bi/few layers in the halo region, and Raman measurements of all the domains that grew in direct contact with the droplets show typical signatures of multilayer WS_2_ (Figure S17). A direct comparison between a SE image, optical image and Raman maps (Figure S18) of the as-grown WS_2_ growth example 4 (see Figure S13) further shows that SE and optical contrasts correlate well with regards to the area of monolayer and few-layer WS_2_. The SE contrast changes thus enable us to also track the nucleation and growth of additional WS_2_ layers.

**Figure 4:**
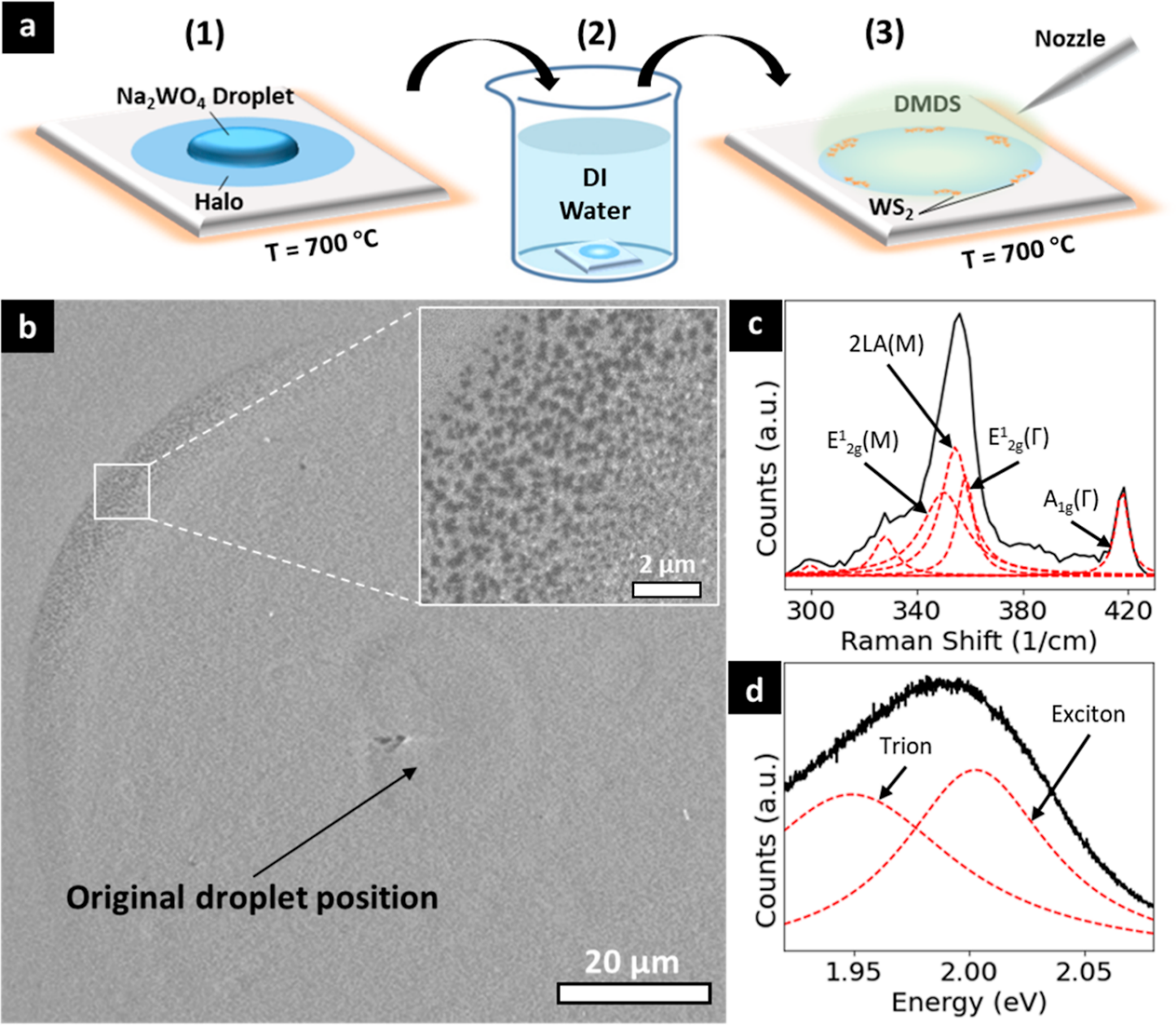
(a) Schematic process flow of “wash-off” experiment. (b) SE image of as-grown WS_2_ on the “washed-off” sample after cooling down, with the original droplet-covered area before washing off the salt being labeled. (c) Raman and (d) PL spectra of the area marked by the white box in (b).

In order to elucidate how the evolution of the halo composition links to W transport, a “wash-off” experiment was performed, schematically illustrated in Figure 4a. The idea thereby is to exploit the water solubility of Na_2_WO_4_ and related salt compositions (Figure 1b) and effectively remove any salt from the sapphire surface. Experimentally (Figure 4a) (1) the Na_2_WO_4_ salt crystals were annealed on sapphire at 700 °C for 10 min, i.e. stage 1 was followed, but then (2) cooled and immersed into 80 °C DI water for 10 min, and (3) the sample was heated up to 700 °C again for a ∼0.1 mbar DMDS exposure. Postwash SEM indeed shows no clearly distinguishable salt particles. However, discernible halo SE contrast regions remain, marking the original positions of the salt crystals. Figure 4b shows a representative SEM image (see Figure S19 for corresponding optical image) of such a washed halo region after stage 2, after having been exposed to DMDS at 700 °C and cooled down. As supported by Raman and PL analysis (Figure 4c,d), we observe small WS_2_ domains particularly in the rim areas of the halo. We can thus conclude that during stage 1 the sapphire had become impregnated in the halo rim with W species that are insoluble in water. The high WS_2_ nucleation density and small WS_2_ domain sizes at stage 2 for these control experiments indicate that there was limited W surface mobility and no further supply of W.

**Figure 5:**
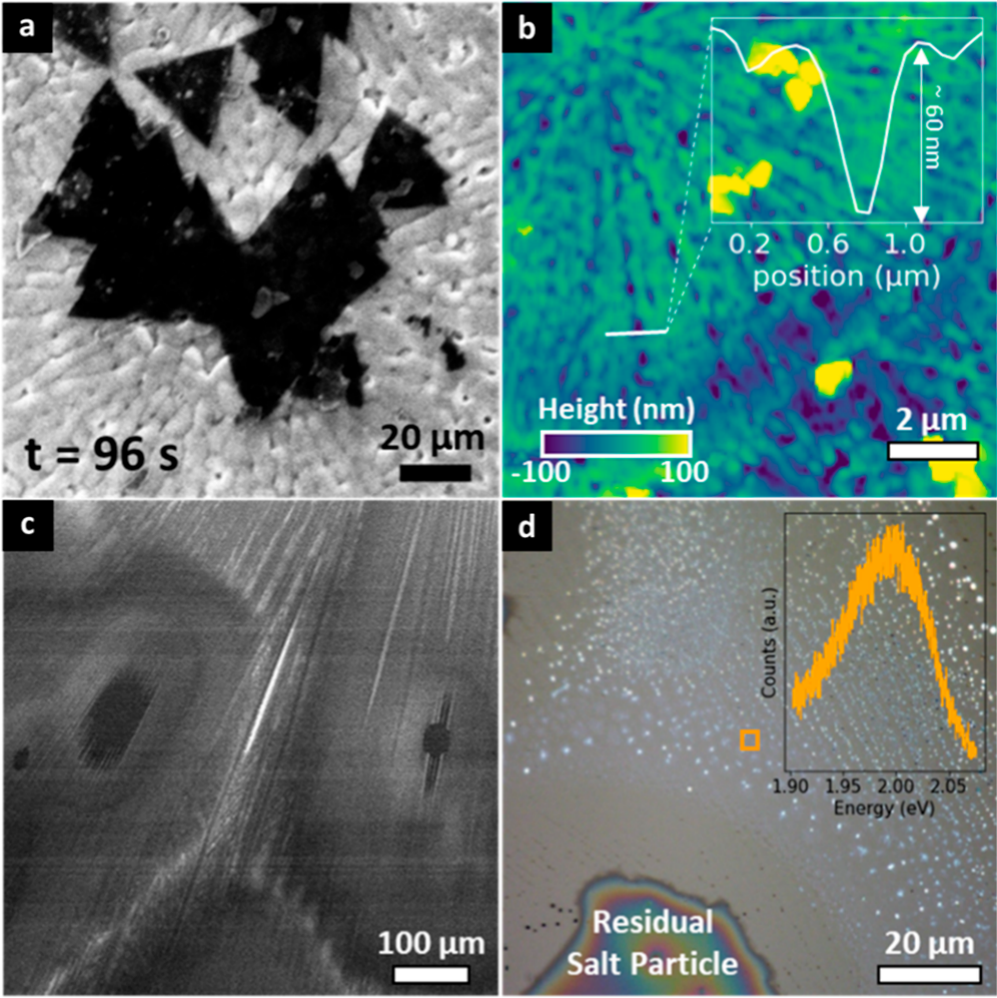
(a) SE image of growing WS_2_ on SiO_2_/Si support at 700 °C under 3 mbar DMDS. (b) Postgrowth AFM map of the SiO_2_/Si support, with the inset line profile showing the depth of an etched trench. (c) SE image of Na_2_WO_4_ droplets on NaOH pretreated sapphire support at *T* = 700 °C. (d) Postgrowth optical image of as-grown WS_2_ on NaOH pretreated sapphire support, grown at 700 °C under 0.1 mbar DMDS.

SiO_2_/Si wafer supports are widely used and it is well referenced that SiO_2_ reacts more readily with alkaline salts than sapphire.^68,69^Figure 5a and the image sequence in Figure S20 show that Na_2_WO_4_ heavily interacts with the SiO_2_ forming significant surface roughness and trenches after the salt melts at 660 °C. Postprocess AFM analysis shows trenches of around 60 nm depth (Figure 5b). The liquid salt efficiently wets and distributes in these trenches at stage 1, leading to darker contrast in the SE images (see Figure S20). At stage 2, this in turn dictates WS_2_ nucleation, feeds layer growth and can also lead to layer dissolution/etching depending on the dynamic redistribution of the remaining liquid salt. We demonstrate similar effects for NaOH pretreated sapphire support (see Experimental Section). NaOH leads to significant sapphire surface corrosion^70^ and trench formation during spin-coating. Figure 5c shows that the liquefied Na_2_WO_4_ salt efficiently wets such pretreated surface (see Figure S21) and rapidly spreads along the trenches. Postgrowth optical data (Figure 5d) shows that this trench pattern dictates WS_2_ nucleation at stage 2, with small domains of mono- to multilayer WS_2_ following this trench pattern. This is further corroborated by experiments on scratched sapphire support (Video S6), where upon melting Na_2_WO_4_ quickly wets and travels in the created indents.

## Discussion

Our operando model system observations show how closely stage 1 salt support interactions dictate the mechanisms of WS_2_ CVD at stage 2. We attempt to rationalize our many process-resolved observations first for the well-defined model system of c-plane sapphire supported Na_2_WO_4_. Stage 1 concerns vacuum annealing. Moving hot (>500 °C) Na_2_WO_4_ particles in situ using a microactuator leaves a clear trace of higher SE yield (see Figure S22). Such support interaction directly underneath the particle reflects a solid–solid or, after melting of the particle, a liquid–solid interface reaction between the hot salt and sapphire. What makes operando SEM so powerful here is that we can observe these support interactions confined to the very surface of sapphire via SE contrast with standard detectors in plan-view at high temperatures and in gas ambient. While macroscopically inert toward alkali metals, sapphire surfaces are known to show localized chemical and physical interactions.^71^ Previous literature also highlights the limited diffusivity^32^ and limited penetration of Na into the bulk of the sapphire, as well as reports Na–O interface layers underneath grown TMD layers.^17^ Any Na reaction and loss of Na_2_O and corresponds to a compositional change of the salt, which closely links to its melting point as highlighted by the eutectic phase diagram (Figure 1b).

**Figure 6:**
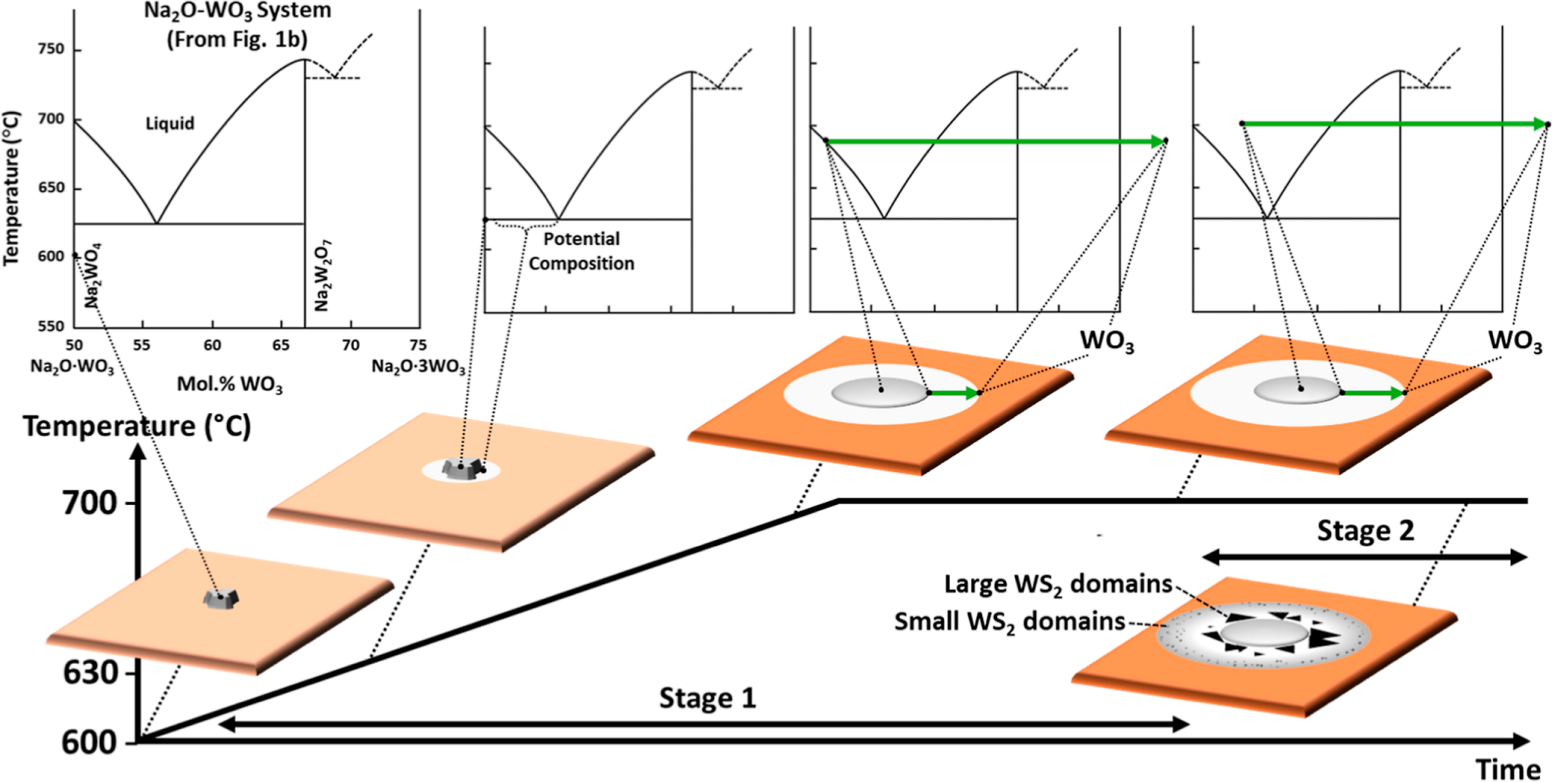
Schematic highlighting the compositional changes of a Na_2_WO_4_ particle/droplet and its halo interface on sapphire at stage 1 and a typical growth scenario at stage 2.

Figure 6 schematically highlights the spatiotemporal compositional changes of a Na_2_WO_4_ particle/droplet and its lateral interface on sapphire. Our data shows that stage 1 annealing above 630 °C leads to a halo formation. This halo area effectively expands vapor, liquid and solid coexistence beyond the liquid salt droplet contact line. Our operando data shows that melting always initiates at the contact with the sapphire and that for the temperature range of approximately 630–690 °C no initial morphological change occurs at the top of the salt particle. We observe a circular halo even in close proximity to the Na_2_WO_4_ particle. We can thus exclude support interactions that are dependent on detailed surface crystallography. The well-defined outer halo interface and its concentric expansion indicate a dominant surface-based transport mechanism. Annealing beyond 700 °C leads to complete salt liquefaction. At this point the lateral droplet footprint significantly increases, reflecting a low wetting angle. The halo formation subsequently continues around the droplet and the bulk salt composition can change to Na_2_W_2_O_7_, as highlighted by postcooling Raman analysis. We find the halo to contain Na and W and postprocess AFM and cross-sectional TEM indicate a nm thick surface layer. The “wash-off” experiment can be consistently rationalized by assuming that nonwater-soluble W oxide only formed at the outer halo rim, while the inner halo and area underneath the particle still had (water-soluble) (1–*x*)Na_2_O·WO_3_ (i.e., Na_2(1–*x*)_WO_4–*x*_) composition. In the outer part of the halo, we propose the concentration can be in the hypereutectic region (right-hand-side of the eutectic point). Concentric SE contrast within the halo as well as ring patterns of post air exposure Na-based particles and of stage 2 WS_2_ nucleation, as well as XPS line scan data are consistent with concentration changes across the halo. While the composition and physical state of the halo are bound to vary, our key stage 1 insight is that driven by sodium tungstate–sapphire interaction the expanding halo represents both Na and W transport into the lateral vicinity of the salt. In other words, an important effect of Na in our model system is that it promotes W surface transport via a reactive Na surface layer formation, and a distinct multiphase interface area.

Stage 2 concerns the DMDS exposure and sulfidation steps that lead to WS_2_ nucleation and growth. Given the high temperatures, DMDS dissociation can occur anywhere on the gas-exposed area of interest on the substrate.^72^ The observed spatial distribution of WS_2_ nucleation thus reflects the W surface distribution at the end of stage 1, and the observed spatiotemporal WS_2_ domain expansion kinetics reflect the further supply of W from the salt source during stage 2. We propose that the significant S solubility of the hot, liquid salt will cause an incubation time to reach the required supersaturation for WS_2_ nucleation. This incubation time will scale with the liquid droplet volume, thus for our model system will be longest for the central salt droplet. A larger S solubility combined with a sizable WS_2_ nucleation barrier will also increase the likelihood of multilayer WS_2_ nucleation, in line with the fact that for given exposure conditions we only observe multilayer WS_2_ on large liquid droplets/areas. Hence the model system does not follow a simple VLS mechanism, where WS_2_ growth would be driven only at the direct liquid droplet interface. We also do not observe mechanisms where the liquid droplet laterally moves as it expels WS_2_ or where the liquid droplet splits and decorates the expanding WS_2_ growth facets.^41,45,48^ Such mechanisms are prevented by the stable wetting of the salt droplet on sapphire and our observation that it is energetically unfavorable for a salt droplet to split up across the range of S exposure conditions and compositional salt changes that dictate surface and interface energies. This indicates that only for support with much lower support interactions, such as on another 2D layer, will a scenario be possible where the growing TMD layer will push the droplet forward.^42^ For conventional support such as sapphire, rather, we find it is the halo interface area around the droplet that is key to controlled WS_2_ formation.

During stage 2, WS_2_ nucleation preferentially occurs across the halo region that formed at stage 1. Concentric ring patterns of WS_2_ nucleation in the initial stage are consistent with the discussed compositional changes across the halo, specifically reflecting the areas where W oxide precipitated. For these small W oxide deposits, supersaturation is quickly achieved. This coincides with the radially inward retreating of the bright SE region. WS_2_ layer expansion requires a further/continuous supply of W at stage 2. We hence find the growth rate of WS_2_ facets to vary inversely with distance from the salt droplet, which is the only source of W in our model system. Closest to the liquid droplet, there remains a region of no WS_2_ nucleation. We propose that this could be due to diffusion and dissolution of S species into the salt droplet, depending on its supersaturation. Eventually WS_2_ growth might also occur in this innermost region around the droplet. Contact of a growing WS_2_ layer with the droplet causes WS_2_ expansion on the liquid, reflecting a locally high concentration of S on the droplet surface, and consistent with a significant barrier to WS_2_ nucleation directly on the liquid surface. WS_2_ layer growth is rate limited by the W supply. Hence the observed growth kinetics and heat maps reflect the W transport during stage 2 originating from the central salt droplet. The inner region around the droplet sees large lateral (>20 μm) monolayer WS_2_ crystal domain sizes, also as the initial WS_2_ nucleation density was lower there. Consistently, the inner region is also where multilayer WS_2_ growth initiates. This region represents the most desirable conditions for monolayer WS_2_ CVD with sufficient yet no excessive supply of both W and S. The adatom mobility of W species is often highlighted as size-limiting for WS_2_ crystal growth in this context.^7,73^ Our model system evidences effective W transport in the halo region in excess of 50 μm, aided by a Na-induced surface layer and partial surface liquefaction. We note that our stage 1 observations refer to high vacuum conditions. The revealed support interactions can be highly dependent on the gas atmosphere used, consistent with the plurality of effects reported for surface-bound Na_2_WO_4_ and salt promotion across the literature.

Hot salt corrosion is significant for SiO_2_ support, with the resulting roughening completely changing the Na_2_WO_4_ wetting and thus distribution of Na and W across the substrate. This is consistent with strong support interactions reported in heterogeneous catalysis for Na_2_WO_4_ on SiO_2_, including surface Si–O–W states.^68,69,74^ While the level of corrosion will be deposition condition dependent, this places a constraint on transfer-free TMD process routes where substrate degradation is to be avoided. This is in addition to the high rates of ion diffusion of alkali metals in SiO_2_, that are well-known in the semiconductor industry to lead to hysteretic and unreliable device performance.^75−77^ In contrast, for transfer-based routes the substrate roughening might ease WS_2_ layer pick-up from the SiO_2_, but WS_2_ purity and defect densities remain a concern. Sapphire does not show such significant corrosive roughening, but for scratched (Video S6) or NaOH etched sapphire (Figure 5c) we observe (micro)trenches to be rapidly wetted and filled with liquid Na_2_WO_4_ as for rough SiO_2_. In these cases, the observed spatiotemporal nucleation and WS_2_ growth behavior comprises growth in the surface layer together with the saturation-precipitation mechanism directly in the liquid. Accordingly, at our conditions, this leads to predominantly multilayer WS_2_ growth when in direct contact to liquid salt, whereas monolayer domains can be found in lateral proximity to the trenches (Figure 5d), consistent with the halo interface region kinetics discussed above.

## Conclusions

Our customized SEM platform enables process-resolved operando observation capability for surface-bound salt-assisted WS_2_ layer CVD. We demonstrate that SE contrast in plan-view at high temperatures and in gas ambient enables detailed tracking not only of spatiotemporal WS_2_ nucleation and 2D crystal layer growth but also of salt-driven support interactions. While such support interactions have to date not received a lot of attention in this field, our results show that they can be of key importance not only in terms of dictating underpinning growth mechanisms but also for future heterogeneous material and process integration. Growth mechanisms like VLS are dictated by droplet wetting, and our data shows that for the model system of individual tungstate salt particles on c-plane sapphire support the wetting behavior is dictated by interfacial film formation and surface eutectic melting. This makes such support interaction extend laterally beyond the contact footprint of the salt, thereby effectively expanding the vapor, liquid and solid coexistence beyond the liquid salt contact line. This provides the dominant Na and W transport paths, and the W transport controls the spatiotemporal WS_2_ growth behavior. For widely used SiO_2_/Si support we find that salt support interactions can be very corrosive resulting in significant surface roughening. This leads to capillary flow of liquid salt, which in turn results in distinctly different spatiotemporal WS_2_ growth behavior. Our insights here on model systems, make a strong case that support interactions should be considered in much more detail for a deeper understanding of salt-assisted TMD CVD and when discussing the opportunities and trade-offs of processing opportunities that salts can bring.

## Experimental Section

### Sample Preparation

The sapphire (*c*-axis, Alfa Aesar) and SiO_2_/Si (200 nm oxide layer) substrates were cleaned by 5 min sonication in acetone, followed by 5 min sonication in isopropanol, and then rinsed in 80 °C DI water for 5 min. Next, the substrates were treated by reactive-ion etching (RIE) with 80 sccm O_2_ and 120 W power for 10 min. Afterward, Na_2_WO_4_·2H_2_O solid particles (Aldrich 99.995% trace metals basis) were spread randomly on the substrate (each/mm^2^ on average), and then the samples were immediately brought to the operando SEM or a low-pressure CVD reactor for the next steps. For spin-coated samples, 2.5 M Na_2_WO_4_ water solution (saturated) was spin-coated on SiO_2_/Si substrates at 1500 rpm, after the RIE treatment. For the NaOH pretreated sample, 0.25 M NaOH water solution was spin-coated on the sapphire substrate at 5000 rpm, after the RIE treatment. The salt particles were then spread on the substrate and brought to the operando SEM.

### SEM Imaging

A high-resolution field emission ZEISS Gemini 300 SEM was used, equipped with a differentially pumped column. Typically, an acceleration voltage of 5 kV was employed, at a working distance of around 13 mm and an aperture size of 30 μm. The Zeiss In-Lens SE detector was used for all recordings shown.

### MS-CVD in Operando SEM

The substrates with the salt particles were loaded in a Kammrath & Weiss 1050 Heating Module in the SEM. The samples were annealed following a typical heating profile shown in Figure S2. The two holding steps were designed to equalize the temperature of the heating stage and the sample. The temperature of the sample was calibrated by the melting point of the Na_2_WO_4_ salt particles. For large Na_2_WO_4_ salt particles (>100 μm), their composition remained to be close to Na_2_WO_4_ upon heating, so the melting point (*T*_m_) of these particles should be 698 °C. We assume the relation between the temperature of the heating stage measured by a thermal couple (*T*_reading_) and the actual temperature of the sample (*T*_real_) is linear and *T*_real_ = 25 °C when *T*_reading_ = 25 °C, hence
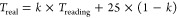
where . DMDS was injected through a tapered quartz nozzle onto the molten salt droplets (or a specific area on a spin-coated sample) to grow WS_2_. The nozzle was made by a Sutter Instrument P-2000 laser-based micropipette puller. Figure S2a,b show top-view and side-view SE images of the nozzle, respectively. Figure S2c shows a Test-Particle Monte Carlo (TPMC) simulation^78^ result of the DMDS pressure distribution on the sample. The molten salt droplets were placed in the highest-pressure region. Since the sample 1 mm away from the highest-pressure region was still under high vacuum, no sulfurization occurred for the droplets in this area. Therefore, on completion of a growth event (i.e., the salt droplet was fully consumed), we moved to an intact droplet to repeat the growth process. Tens of growth events can be observed on an 8 × 8 mm^2^ substrate. The sample was finally cooled in vacuum to room temperature at a rate of approximately 20 °C/min.

### Post-Process Characterizations

#### Sample Storage

For all the postprocess (stage 1 or 2) characterization, the samples were vacuum-sealed immediately after being cooled down and taken out from the SEM to minimize the air-exposure. The samples were typically stored in vacuum for 3–7 days before the analysis.

#### Cross-Sectional FIB and TEM

Cross-sectional FIB lamellae were fabricated using an FEI Helios NanoLab 660 dual beam FIB. The samples were coated with approximately 20 nm of carbon prior to FIB processing to reduce charging and protect the sample. Target area was coated with 2 μm of Pt via electron beam and ion beam deposition, before a bulk mill at 30 kV and sample thinning at voltages down to 5 kV to achieve electron transparency. TEM images were obtained with an FEI Talos F200A TEM at 200 kV with four FEI Super-X EDS detectors. Experimental conditions for the STEM based EDS mapping were as follows: estimated probe currents and probe diameters were ∼1.5 nA and ∼1 nm respectively, with a 25 μs dwell time. The elemental maps were processed using the Velox Software from ThermoFisher Scientific, and a 5 px average was applied to the images.

#### XPS

XPS Analysis was performed using a Thermo NEXSA G2 XPS fitted with a monochromated Al kα X-ray source (1486.7 eV), a spherical sector analyzer and 3 multichannel resistive plate, 128 channel delay line detectors. All data was recorded at 19.2 W and an X-ray beam size of 20 × 10 μm. Survey scans were recorded at a pass energy of 200 eV, and high-resolution scans recorded at a pass energy of 50 eV. Electronic charge neutralization was achieved using an ion source (Thermo Scientific FG-03). Ion gun current = 150 μA. Ion gun voltage = 40 V. All sample data was recorded at a pressure below 10^–8^ Torr and a room temperature of 294 K. The spectra were calibrated with Na 1s peaks to compensate differential charging. Data was analyzed using CasaXPS v2.3.26rev1.0N. Peaks were fit with a Shirley background prior to component analysis. Lineshapes of LA(1.53,243) were used to fit components.

#### AFM

AFM measurements were performed using a MFP-3D AFM system (Asylum/Oxford Instruments) in tapping mode. The tip used was a Tap300Al-G from BudgetSensors (resonance frequency = 300 kHz).

#### SEM Based EDS

A ZEISS Merlin SEM was used for the EDS included in this work. The acceleration voltage of the electron beam (e-beam) was set to 2 kV. The probe current was ∼800 pA. A long signal processing time (∼2 min) was used to maximize the energy resolution of the spectra. The EDS detector used was an Oxford Instruments X-Max Extreme windowless detector running acquisition/analysis software AZtec v6.1. The detector geometry is optimized for a short working distance of 4.5 mm.

### Raman and PL Spectroscopy

Raman and PL spectra were measured using a Renishaw inVia Raman microscope at room temperature. A 532 nm laser (20 mW), an 1800 lines/mm grating and a 100× objective were used for the measurements. For the Raman measurements, the exposure time, laser power, and integration time were 1 s, 1% power, and 1 time, respectively. For PL measurements, the exposure time, laser power, and integration time were 1 s, 0.1% power, and 1 time (0.1 s, 1% power, and 1 time for mapping), respectively. For both Figure 4c,d, the exposure time, laser power, and integration time were 5 s, 5% power, and 1 time, respectively. All relevant peaks in the Raman and PL spectra were fitted with a Lorentzian distribution using python codes.

### Ex situ Stage 1 Annealing

A low-pressure cold-wall CVD reactor was used for comparative ex-situ sample annealing. The substrate with the salt particles was loaded on a graphite stage heated from the back with a continuous wave IR laser (808 nm) with a top-hat beam shaper. The chamber pressure was lowered to below 10^–5^ mbar after sample loading, and then the power of the laser was increased to 40 W at a rate of 1.2 W/min. The sample temperature was measured with an IR pyrometer. The sample temperature increased roughly linearly to ∼800 °C at a rate of around 25 °C/min. The sample was annealed at ∼800 °C for 10 min and finally cooled to room temperature at ∼50 °C/min. The sample was then brought to the SEM immediately for postprocess imaging.

## Supporting Information Available

Operando SEM videos, their description and additional data on quartz μ-I, process profiles and sample analysis, including further TEM, XPS, SEM, AFM, STEM and SEM based EDS results, and correlative optical/Raman/PL mapping (PDF)
Salt Particle Melting (MP4)
Growth example 1 (MP4)
Growth example 2 (MP4)
Growth example 3 (MP4)
Growth example 4 (MP4)
Salt Melting on Scratch (MP4)

## References

[ref1] Lemme M. C.; Akinwande D.; Huyghebaert C.; Stampfer C. 2D Materials for Future Heterogeneous Electronics. Nat. Commun. 2022, 13 (1), 1392 10.1038/s41467-022-29001-4. 35296657 PMC8927416

[ref2] International Roadmap for Devices and Systems (IRDSTM), 2022 Edition, Beyond CMOS and Emerging Research Materials; 2022. https://irds.ieee.org/images/files/pdf/2022/2022IRDS_BC.pdf (accessed 2024–04–18).

[ref3] O’Brien K. P.; Naylor C. H.; Dorow C.; Maxey K.; Penumatcha A. V.; Vyatskikh A.; Zhong T.; Kitamura A.; Lee S.; Rogan C.; Mortelmans W.; Kavrik M. S.; Steinhardt R.; Buragohain P.; Dutta S.; Tronic T.; Clendenning S.; Fischer P.; Putna E. S.; Radosavljevic M.; Metz M.; Avci U. Process Integration and Future Outlook of 2D Transistors. Nat. Commun. 2023, 14 (1), 6400 10.1038/s41467-023-41779-5. 37828036 PMC10570266

[ref4] Kang K.; Xie S.; Huang L.; Han Y.; Huang P. Y.; Mak K. F.; Kim C.-J.; Muller D.; Park J. High-Mobility Three-Atom-Thick Semiconducting Films with Wafer-Scale Homogeneity. Nature 2015, 520 (7549), 656–660. 10.1038/nature14417. 25925478

[ref5] Lee Y.-H.; Zhang X.-Q.; Zhang W.; Chang M.-T.; Lin C.-T.; Chang K.-D.; Yu Y.-C.; Wang J. T.-W.; Chang C.-S.; Li L.-J.; Lin T.-W. Synthesis of Large-Area MoS2 Atomic Layers with Chemical Vapor Deposition. Adv. Mater. 2012, 24 (17), 2320–2325. 10.1002/adma.201104798. 22467187

[ref6] Choudhury T. H.; Simchi H.; Boichot R.; Chubarov M.; Mohney S. E.; Redwing J. M. Chalcogen Precursor Effect on Cold-Wall Gas-Source Chemical Vapor Deposition Growth of WS2. Cryst. Growth Des. 2018, 18 (8), 4357–4364. 10.1021/acs.cgd.8b00306.

[ref7] Zhang X.; Choudhury T. H.; Chubarov M.; Xiang Y.; Jariwala B.; Zhang F.; Alem N.; Wang G.-C.; Robinson J. A.; Redwing J. M. Diffusion-Controlled Epitaxy of Large Area Coalesced WSe2Monolayers on Sapphire. Nano Lett. 2018, 18 (2), 1049–1056. 10.1021/acs.nanolett.7b04521. 29342357

[ref8] Chubarov M.; Choudhury T. H.; Hickey D. R.; Bachu S.; Zhang T.; Sebastian A.; Bansal A.; Zhu H.; Trainor N.; Das S.; Terrones M.; Alem N.; Redwing J. M. Wafer-Scale Epitaxial Growth of Unidirectional WS2Monolayers on Sapphire. ACS Nano 2021, 15 (2), 2532–2541. 10.1021/acsnano.0c06750. 33450158

[ref9] Eichfeld S. M.; Hossain L.; Lin Y.-C.; Piasecki A. F.; Kupp B.; Birdwell A. G.; Burke R. A.; Lu N.; Peng X.; Li J.; Azcatl A.; McDonnell S.; Wallace R. M.; Kim M. J.; Mayer T. S.; Redwing J. M.; Robinson J. A. Highly Scalable, Atomically Thin WSe2 Grown via Metal–Organic Chemical Vapor Deposition. ACS Nano 2015, 9 (2), 2080–2087. 10.1021/nn5073286. 25625184

[ref10] Voronenkov V.; Groven B.; Medina Silva H.; Morin P.; De Gendt S. Guiding Principles for the Design of a Chemical Vapor Deposition Process for Highly Crystalline Transition Metal Dichalcogenides. Phys. Status Solidi A 2024, 221, 2300943 10.1002/pssa.202300943.

[ref11] Kandybka I.; Groven B.; Medina Silva H.; Sergeant S.; Nalin Mehta A.; Koylan S.; Shi Y.; Banerjee S.; Morin P.; Delabie A. Chemical Vapor Deposition of a Single-Crystalline MoS2Monolayer through Anisotropic 2D Crystal Growth on Stepped Sapphire Surface. ACS Nano 2024, 18 (4), 3173–3186. 10.1021/acsnano.3c09364. 38235963

[ref12] Lei J.; Xie Y.; Kutana A.; Bets K. V.; Yakobson B. I. Salt-Assisted MoS2 Growth: Molecular Mechanisms from the First Principles. J. Am. Chem. Soc. 2022, 144 (16), 7497–7503. 10.1021/jacs.2c02497. 35427122

[ref13] Dziobek-Garrett R.; Hilliard S.; Sriramineni S.; Ambrozaite O.; Zhu Y.; Hudak B. M.; Brintlinger T. H.; Chowdhury T.; Kempa T. J. Controlling Morphology and Excitonic Disorder in Monolayer WSe2 Grown by Salt-Assisted CVD Methods. ACS Nanosci. Au 2023, 3 (6), 441–450. 10.1021/acsnanoscienceau.3c00028. 38144700 PMC10740127

[ref14] Zhou J.; Lin J.; Huang X.; Zhou Y.; Chen Y.; Xia J.; Wang H.; Xie Y.; Yu H.; Lei J.; Wu D.; Liu F.; Fu Q.; Zeng Q.; Hsu C.-H.; Yang C.; Lu L.; Yu T.; Shen Z.; Lin H.; Yakobson B. I.; Liu Q.; Suenaga K.; Liu G.; Liu Z. A Library of Atomically Thin Metal Chalcogenides. Nature 2018, 556 (7701), 355–359. 10.1038/s41586-018-0008-3. 29670263

[ref15] Kim H.; Ovchinnikov D.; Deiana D.; Unuchek D.; Kis A. Suppressing Nucleation in Metal–Organic Chemical Vapor Deposition of MoS2Monolayers by Alkali Metal Halides. Nano Lett. 2017, 17 (8), 5056–5063. 10.1021/acs.nanolett.7b02311. 28700239

[ref16] Chang M.-C.; Ho P.-H.; Tseng M.-F.; Lin F.-Y.; Hou C.-H.; Lin I.-K.; Wang H.; Huang P.-P.; Chiang C.-H.; Yang Y.-C.; Wang I.-T.; Du H.-Y.; Wen C.-Y.; Shyue J.-J.; Chen C.-W.; Chen K.-H.; Chiu P.-W.; Chen L.-C. Fast Growth of Large-Grain and Continuous MoS2 Films through a Self-Capping Vapor-Liquid-Solid Method. Nat. Commun. 2020, 11 (1), 3682 10.1038/s41467-020-17517-6. 32703950 PMC7378841

[ref17] Zhang K.; Bersch B. M.; Zhang F.; Briggs N. C.; Subramanian S.; Xu K.; Chubarov M.; Wang K.; Lerach J. O.; Redwing J. M.; Fullerton-Shirey S. K.; Terrones M.; Robinson J. A. Considerations for Utilizing Sodium Chloride in Epitaxial Molybdenum Disulfide. ACS Appl. Mater. Interfaces 2018, 10 (47), 40831–40837. 10.1021/acsami.8b16374. 30384598

[ref18] Wang Z.; Xie Y.; Wang H.; Wu R.; Nan T.; Zhan Y.; Sun J.; Jiang T.; Zhao Y.; Lei Y.; Yang M.; Wang W.; Zhu Q.; Ma X.; Hao Y. NaCl-Assisted One-Step Growth of MoS2–WS2 in-Plane Heterostructures. Nanotechnology 2017, 28 (32), 325602 10.1088/1361-6528/aa6f01. 28718451

[ref19] Zhu J.; Park J.-H.; Vitale S. A.; Ge W.; Jung G. S.; Wang J.; Mohamed M.; Zhang T.; Ashok M.; Xue M.; Zheng X.; Wang Z.; Hansryd J.; Chandrakasan A. P.; Kong J.; Palacios T. Low-Thermal-Budget Synthesis of Monolayer Molybdenum Disulfide for Silicon Back-End-of-Line Integration on a 200 Mm Platform. Nat. Nanotechnol. 2023, 18 (5), 456–463. 10.1038/s41565-023-01375-6. 37106051

[ref20] Li S.; Lin Y.-C.; Liu X.-Y.; Hu Z.; Wu J.; Nakajima H.; Liu S.; Okazaki T.; Chen W.; Minari T.; Sakuma Y.; Tsukagoshi K.; Suenaga K.; Taniguchi T.; Osada M. Wafer-Scale and Deterministic Patterned Growth of Monolayer MoS2via Vapor–Liquid–Solid Method. Nanoscale 2019, 11 (34), 16122–16129. 10.1039/C9NR04612G. 31433425

[ref21] Qin Z.; Loh L.; Wang J.; Xu X.; Zhang Q.; Haas B.; Alvarez C.; Okuno H.; Yong J. Z.; Schultz T.; Koch N.; Dan J.; Pennycook S. J.; Zeng D.; Bosman M.; Eda G. Growth of Nb-Doped Monolayer WS2 by Liquid-Phase Precursor Mixing. ACS Nano 2019, 13 (9), 10768–10775. 10.1021/acsnano.9b05574. 31491079

[ref22] Cun H.; Macha M.; Kim H.; Liu K.; Zhao Y.; LaGrange T.; Kis A.; Radenovic A. Wafer-Scale MOCVD Growth of Monolayer MoS2 on Sapphire and SiO2. Nano Res. 2019, 12 (10), 2646–2652. 10.1007/s12274-019-2502-9.

[ref23] Liu H.; Qi G.; Tang C.; Chen M.; Chen Y.; Shu Z.; Xiang H.; Jin Y.; Wang S.; Li H.; Ouzounian M.; Hu T. S.; Duan H.; Li S.; Han Z.; Liu S. Growth of Large-Area Homogeneous Monolayer Transition-Metal Disulfides via a Molten Liquid Intermediate Process. ACS Appl. Mater. Interfaces 2020, 12 (11), 13174–13181. 10.1021/acsami.9b22397. 32103663

[ref24] Zuo Y.; Yu W.; Liu C.; Cheng X.; Qiao R.; Liang J.; Zhou X.; Wang J.; Wu M.; Zhao Y.; Gao P.; Wu S.; Sun Z.; Liu K.; Bai X.; Liu Z. Optical Fibres with Embedded Two-Dimensional Materials for Ultrahigh Nonlinearity. Nat. Nanotechnol. 2020, 15 (12), 987–991. 10.1038/s41565-020-0770-x. 32958935

[ref25] Fan S.; Yun S. J.; Yu W. J.; Lee Y. H. Tailoring Quantum Tunneling in a Vanadium-Doped WSe2/SnSe2 Heterostructure. Advanced Science 2020, 7 (3), 1902751 10.1002/advs.201902751. 32042571 PMC7001641

[ref26] Yun S. J.; Duong D. L.; Ha D. M.; Singh K.; Phan T. L.; Choi W.; Kim Y.-M.; Lee Y. H. Ferromagnetic Order at Room Temperature in Monolayer WSe2 Semiconductor via Vanadium Dopant. Advanced Science 2020, 7 (9), 1903076 10.1002/advs.201903076. 32382479 PMC7201245

[ref27] Vu V. T.; Vu T. T. H.; Phan T. L.; Kang W. T.; Kim Y. R.; Tran M. D.; Nguyen H. T. T.; Lee Y. H.; Yu W. J. One-Step Synthesis of NbSe2/Nb-Doped-WSe2Metal/Doped-Semiconductor van Der Waals Heterostructures for Doping Controlled Ohmic Contact. ACS Nano 2021, 15 (8), 13031–13040. 10.1021/acsnano.1c02038. 34350752

[ref28] Li S.; Hong J.; Gao B.; Lin Y.-C.; Lim H. E.; Lu X.; Wu J.; Liu S.; Tateyama Y.; Sakuma Y.; Tsukagoshi K.; Suenaga K.; Taniguchi T. Tunable Doping of Rhenium and Vanadium into Transition Metal Dichalcogenides for Two-Dimensional Electronics. Advanced Science 2021, 8 (11), 2004438 10.1002/advs.202004438. 34105285 PMC8188190

[ref29] Han S. W.; Yun W. S.; Woo W. J.; Kim H.; Park J.; Hwang Y. H.; Nguyen T. K.; Le C. T.; Kim Y. S.; Kang M.; Ahn C. W.; Hong S. C. Interface Defect Engineering of a Large-Scale CVD-Grown MoS2Monolayer via Residual Sodium at the SiO2/Si Substrate. Adv. Mater. Interfaces 2021, 8 (14), 2100428 10.1002/admi.202100428.

[ref30] Chang Y.-P.; Li W.-B.; Yang Y.-C.; Lu H.-L.; Lin M.-F.; Chiu P.-W.; Lin K.-I. Oxidation and Degradation of WS2Monolayers Grown by NaCl-Assisted Chemical Vapor Deposition: Mechanism and Prevention. Nanoscale 2021, 13 (39), 16629–16640. 10.1039/D1NR04809K. 34586136

[ref31] Hu S.; Finklea H.; Liu X. A Review on Molten Sulfate Salts Induced Hot Corrosion. J. Mater. Sci. Technol. 2021, 90, 243–254. 10.1016/j.jmst.2021.03.013.

[ref32] Doremus R. H. Diffusion in Alumina. J. Appl. Phys. 2006, 100 (10), 101301 10.1063/1.2393012.

[ref33] Yang P.; Zou X.; Zhang Z.; Hong M.; Shi J.; Chen S.; Shu J.; Zhao L.; Jiang S.; Zhou X.; Huan Y.; Xie C.; Gao P.; Chen Q.; Zhang Q.; Liu Z.; Zhang Y. Batch Production of 6-Inch Uniform Monolayer Molybdenum Disulfide Catalyzed by Sodium in Glass. Nat. Commun. 2018, 9 (1), 979 10.1038/s41467-018-03388-5. 29515118 PMC5841402

[ref34] Li X.; Kahn E.; Chen G.; Sang X.; Lei J.; Passarello D.; Oyedele A. D.; Zakhidov D.; Chen K.-W.; Chen Y.-X.; Hsieh S.-H.; Fujisawa K.; Unocic R. R.; Xiao K.; Salleo A.; Toney M. F.; Chen C.-H.; Kaxiras E.; Terrones M.; Yakobson B. I.; Harutyunyan A. R. Surfactant-Mediated Growth and Patterning of Atomically Thin Transition Metal Dichalcogenides. ACS Nano 2020, 14 (6), 6570–6581. 10.1021/acsnano.0c00132. 32338865

[ref35] Li S.; Lin Y.-C.; Hong J.; Gao B.; Lim H. E.; Yang X.; Liu S.; Tateyama Y.; Tsukagoshi K.; Sakuma Y.; Suenaga K.; Taniguchi T. Mixed-Salt Enhanced Chemical Vapor Deposition of Two-Dimensional Transition Metal Dichalcogenides. Chem. Mater. 2021, 33 (18), 7301–7308. 10.1021/acs.chemmater.1c01652.

[ref36] Kim M.; Seo J.; Kim J.; Moon J. S.; Lee J.; Kim J.-H.; Kang J.; Park H. High-Crystalline Monolayer Transition Metal Dichalcogenides Films for Wafer-Scale Electronics. ACS Nano 2021, 15 (2), 3038–3046. 10.1021/acsnano.0c09430. 33512141

[ref37] Ji Q.; Su C.; Mao N.; Tian X.; Idrobo J.-C.; Miao J.; Tisdale W. A.; Zettl A.; Li J.; Kong J. Revealing the Brønsted-Evans-Polanyi Relation in Halide-Activated Fast MoS _2_ Growth toward Millimeter-Sized 2D Crystals. Sci. Adv. 2021, 7 (44), eabj3274 10.1126/sciadv.abj3274. 34705498 PMC8550239

[ref38] Song J.-G.; Hee Ryu G.; Kim Y.; Je Woo W.; Yong Ko K.; Kim Y.; Lee C.; Oh I.-K.; Park J.; Lee Z.; Kim H. Catalytic Chemical Vapor Deposition of Large-Area Uniform Two-Dimensional Molybdenum Disulfide Using Sodium Chloride. Nanotechnology 2017, 28 (46), 465103 10.1088/1361-6528/aa8f15. 29059049

[ref39] Wang P.; Lei J.; Qu J.; Cao S.; Jiang H.; He M.; Shi H.; Sun X.; Gao B.; Liu W. Mechanism of Alkali Metal Compound-Promoted Growth of Monolayer MoS2: Eutectic Intermediates. Chem. Mater. 2019, 31 (3), 873–880. 10.1021/acs.chemmater.8b04022.

[ref40] Ma L.; Zhu J.; Li W.; Huang R.; Wang X.; Guo J.; Choi J.-H.; Lou Y.; Wang D.; Zou G. Immobilized Precursor Particle Driven Growth of Centimeter-Sized MoTe2Monolayer. J. Am. Chem. Soc. 2021, 143 (33), 13314–13324. 10.1021/jacs.1c06250. 34375083

[ref41] Huang L.; Thi Q. H.; Zheng F.; Chen X.; Chu Y. W.; Lee C.-S.; Zhao J.; Ly T. H. Catalyzed Kinetic Growth in Two-Dimensional MoS2. J. Am. Chem. Soc. 2020, 142 (30), 13130–13135. 10.1021/jacs.0c05057. 32614184

[ref42] Li S.; Lin Y.-C.; Zhao W.; Wu J.; Wang Z.; Hu Z.; Shen Y.; Tang D.-M.; Wang J.; Zhang Q.; Zhu H.; Chu L.; Zhao W.; Liu C.; Sun Z.; Taniguchi T.; Osada M.; Chen W.; Xu Q.-H.; Wee A. T. S.; Suenaga K.; Ding F.; Eda G. Vapour–Liquid–Solid Growth of Monolayer MoS2 Nanoribbons. Nat. Mater. 2018, 17 (6), 535–542. 10.1038/s41563-018-0055-z. 29686277

[ref43] Rasouli H. R.; Mehmood N.; Çakıroğlu O.; Kasırga T. S. Real Time Optical Observation and Control of Atomically Thin Transition Metal Dichalcogenide Synthesis. Nanoscale 2019, 11 (15), 7317–7323. 10.1039/C9NR00614A. 30938382

[ref44] Jiang D.; Wang X.; Chen R.; Sun J.; Kang H.; Ji D.; Liu Y.; Wei D. Self-Expanding Molten Salt-Driven Growth of Patterned Transition-Metal Dichalcogenide Crystals. J. Am. Chem. Soc. 2022, 144 (19), 8746–8755. 10.1021/jacs.2c02518. 35508181

[ref45] Yang S.; Wu J.; Wang C.; Yan H.; Han L.; Feng J.; Zhang B.; Li D.; Yu G.; Luo B. Molten-Droplet-Driven Growth of MoS2 Flakes with Controllable Morphology Transition for Hydrogen Evolution Reactions. Dalton Trans. 2022, 51 (35), 13351–13360. 10.1039/D2DT02066A. 35984420

[ref46] Lee J.; Shin N. Toward an Understanding of the Mechanism of Mixed-Salt-Mediated CVD Growth of MoSe2. Appl. Phys. Lett. 2023, 123 (18), 181902 10.1063/5.0165703.

[ref47] He S.; Cheng Z.; Xin D.; Zhang X.; Zhang R.; Zhang X.; Liu Z.; Zhang S.; Xia M. Manipulation of the 1T-MoS2 Domain in a 2H-MoS2Main Phase Induced by V-Doping via a CVD Vapor–Liquid–Solid Mechanism. CrystEngComm 2022, 24 (48), 8517–8524. 10.1039/D2CE01305C.

[ref48] Yang S.; Wang C.; Wu J.; Yan H.; Wang G.; Feng J.; Zhang B.; Li D.; Booth T. J.; Bøggild P.; Yu G.; Luo B. Self-Relaxation Vapor-Liquid-Solid Growth of Two-Dimensional Transition Metal Dichalcogenides with Loose Interface. Appl. Surf. Sci. 2023, 613, 156019 10.1016/j.apsusc.2022.156019.

[ref49] Wu J.; Zhang Y.; Jia Z.; Ma Z.; Song J. Study on the Morphological Mechanism of MoS2 Growth by NaCl-Assisted Chemical Vapor Deposition. ChemistrySelect 2023, 8 (35), e202301599 10.1002/slct.202301599.

[ref50] Momeni K.; Ji Y.; Wang Y.; Paul S.; Neshani S.; Yilmaz D. E.; Shin Y. K.; Zhang D.; Jiang J.-W.; Park H. S.; Sinnott S.; van Duin A.; Crespi V.; Chen L.-Q. Multiscale Computational Understanding and Growth of 2D Materials: A Review. NPJ. Comput. Mater. 2020, 6 (1), 22 10.1038/s41524-020-0280-2.

[ref51] Hofmann S.; Sharma R.; Wirth C. T.; Cervantes-Sodi F.; Ducati C.; Kasama T.; Dunin-Borkowski R. E.; Drucker J.; Bennett P.; Robertson J. Ledge-Flow-Controlled Catalyst Interface Dynamics during Si Nanowire Growth. Nat. Mater. 2008, 7 (5), 372–375. 10.1038/nmat2140. 18327262

[ref52] Zhang J.; Zhai T.; Arifurrahman F.; Wang Y.; Hitt A.; He Z.; Ai Q.; Liu Y.; Lin C.-Y.; Zhu Y.; Tang M.; Lou J. Toward Controlled Synthesis of 2D Crystals by CVD: Learning from the Real-Time Crystal Morphology Evolutions. Nano Lett. 2024, 24 (8), 2465–2472. 10.1021/acs.nanolett.3c04016. 38349857

[ref53] Fan Y.; Nakanishi K.; Veigang-Radulescu V. P.; Mizuta R.; Stewart J. C.; Swallow J. E. N.; Dearle A. E.; Burton O. J.; Alexander-Webber J. A.; Ferrer P.; Held G.; Brennan B.; Pollard A. J.; Weatherup R. S.; Hofmann S. Understanding Metal Organic Chemical Vapour Deposition of Monolayer WS2: The Enhancing Role of Au Substrate for Simple Organosulfur Precursors. Nanoscale 2020, 12 (43), 22234–22244. 10.1039/D0NR06459A. 33141137

[ref54] Xue H.; Wu G.; Zhao B.; Wang D.; Wu X.; Hu Z. High-Temperature In Situ Investigation of Chemical Vapor Deposition to Reveal Growth Mechanisms of Monolayer Molybdenum Disulfide. ACS Appl. Electron. Mater. 2020, 2 (7), 1925–1933. 10.1021/acsaelm.0c00231.

[ref55] Yamdagni R.; Pupp C.; Porter R. F. Mass Spectrometric Study of the Evaporation of Lithium and Sodium Molybdates and Tungstates. J. Inorg. Nucl. Chem. 1970, 32 (11), 3509–3523. 10.1016/0022-1902(70)80159-2.

[ref56] Chang L. L. Y.; Sachdev S. Alkali Tungstates: Stability Relations in the Systems A2O WO3-WO3. J. Am. Ceram. Soc. 1975, 58 (7–8), 267–270. 10.1111/j.1151-2916.1975.tb11472.x.

[ref57] Mann M.; Shter G. E.; Reisner G. M.; Grader G. S. Synthesis of Tungsten Bronze Powder and Determination of Its Composition. J. Mater. Sci. 2007, 42 (3), 1010–1018. 10.1007/s10853-006-1384-x.

[ref58] Janz G. J. Thermodynamic and Transport Properties for Molten Salts: Correlation Equations for Critically Evaluated Density, Surface Tension, Electrical Conductance, and Viscosity Data; American Chemical Society and the American Institute of Physics for the National Bureau of Standards, 1988.

[ref59] Elkins T. W.; Hagelin-Weaver H. E. Characterization of Mn–Na2WO4/SiO2 and Mn–Na2WO4/MgO Catalysts for the Oxidative Coupling of Methane. Appl. Catal., A 2015, 497, 96–106. 10.1016/j.apcata.2015.02.040.

[ref60] Wertheim G. K.; Campagna M.; Chazalviel J.-N.; Buchanan D. N. E.; Shanks H. R. Electronic Structure of Tetragonal Tungsten Bronzes and Electrochromic Oxides. Appl. Phys. 1977, 13 (3), 225–230. 10.1007/BF00882885.

[ref61] Fertig M. P.; Dirksen C.; Schulz M.; Stelter M. Humidity-Induced Degradation of Lithium-Stabilized Sodium-Beta Alumina Solid Electrolytes. Batteries 2022, 8 (9), 103 10.3390/batteries8090103.

[ref62] Okada M.; Okada N.; Chang W.-H.; Endo T.; Ando A.; Shimizu T.; Kubo T.; Miyata Y.; Irisawa T. Gas-Source CVD Growth of Atomic Layered WS2 from WF6 and H2S Precursors with High Grain Size Uniformity. Sci. Rep. 2019, 9 (1), 17678 10.1038/s41598-019-54049-6. 31776373 PMC6881408

[ref63] Chen Y. Growth of a Large, Single-Crystalline WS2Monolayer for High-Performance Photodetectors by Chemical Vapor Deposition. Micromachines 2021, 12 (2), 137 10.3390/mi12020137. 33514063 PMC7911554

[ref64] Berkdemir A.; Gutiérrez H. R.; Botello-Méndez A. R.; Perea-López N.; Elías A. L.; Chia C.-I.; Wang B.; Crespi V. H.; López-Urías F.; Charlier J.-C.; Terrones H.; Terrones M. Identification of Individual and Few Layers of WS2 Using Raman Spectroscopy. Sci. Rep. 2013, 3 (1), 1755 10.1038/srep01755.

[ref65] McCreary K. M.; Hanbicki A. T.; Singh S.; Kawakami R. K.; Jernigan G. G.; Ishigami M.; Ng A.; Brintlinger T. H.; Stroud R. M.; Jonker B. T. The Effect of Preparation Conditions on Raman and Photoluminescence of Monolayer WS2. Sci. Rep. 2016, 6 (1), 35154 10.1038/srep35154. 27752042 PMC5067492

[ref66] Zhou Y.; Fox D. S.; Maguire P.; O’Connell R.; Masters R.; Rodenburg C.; Wu H.; Dapor M.; Chen Y.; Zhang H. Quantitative Secondary Electron Imaging for Work Function Extraction at Atomic Level and Layer Identification of Graphene. Sci. Rep. 2016, 6 (1), 21045 10.1038/srep21045. 26878907 PMC4754635

[ref67] Shihommatsu K.; Takahashi J.; Momiuchi Y.; Hoshi Y.; Kato H.; Homma Y. Formation Mechanism of Secondary Electron Contrast of Graphene Layers on a Metal Substrate. ACS Omega 2017, 2 (11), 7831–7836. 10.1021/acsomega.7b01550. 31457340 PMC6645151

[ref68] Jiang Z. C.; Yu C. J.; Fang X. P.; Li S. B.; Wang H. L. Oxide/Support Interaction and Surface Reconstruction in the Sodium Tungstate(Na2WO4)/Silica System. J. Phys. Chem. 1993, 97 (49), 12870–12875. 10.1021/j100151a038.

[ref69] Yildiz M.; Aksu Y.; Simon U.; Otremba T.; Kailasam K.; Göbel C.; Girgsdies F.; Görke O.; Rosowski F.; Thomas A.; Schomäcker R.; Arndt S. Silica Material Variation for the MnxOy-Na2WO4/SiO2. Appl. Catal., A 2016, 525, 168–179. 10.1016/j.apcata.2016.06.034.

[ref70] Hu Y.; Yuan B.; Cheng F.; Hu X. NaOH Etching and Resin Pre-Coating Treatments for Stronger Adhesive Bonding between CFRP and Aluminium Alloy. Compos B Eng. 2019, 178, 107478 10.1016/j.compositesb.2019.107478.

[ref71] Rodriguez J. A.; Kuhn M.; Hrbek J. Interaction of Silver, Cesium, and Zinc with Alumina Surfaces: Thermal Desorption and Photoemission Studies. J. Phys. Chem. 1996, 100 (46), 18240–18248. 10.1021/jp962195w.

[ref72] Vandeputte A. G.; Reyniers M.-F.; Marin G. B. Theoretical Study of the Thermal Decomposition of Dimethyl Disulfide. J. Phys. Chem. A 2010, 114 (39), 10531–10549. 10.1021/jp103357z. 20843049

[ref73] Tang S.; Grundmann A.; Fiadziushkin H.; Wang Z.; Hoffmann-Eifert S.; Ghiami A.; Debald A.; Heuken M.; Vescan A.; Kalisch H. Migration-Enhanced Metal–Organic Chemical Vapor Deposition of Wafer-Scale Fully Coalesced WS2 and WSe2Monolayers. Cryst. Growth Des. 2023, 23 (3), 1547–1558. 10.1021/acs.cgd.2c01134.

[ref74] Martín C.; Malet P.; Solana G.; Rives V. Structural Analysis of Silica-Supported Tungstates. J. Phys. Chem. B 1998, 102 (15), 2759–2768. 10.1021/jp980614e.

[ref75] Snow E. H.; Grove A. S.; Deal B. E.; Sah C. T. Ion Transport Phenomena in Insulating Films. J. Appl. Phys. 1965, 36 (5), 1664–1673. 10.1063/1.1703105.

[ref76] Yon E.; Ko W. H.; Kuper A. B. Sodium Distribution in Thermal Oxide on Silicon by Radiochemical and MOS Analysis. IEEE Trans. Electron Devices 1966, ED-13 (2), 276–280. 10.1109/T-ED.1966.15680.

[ref77] Krivec S.; Buchmayr M.; Detzel T.; Froemling T.; Fleig J.; Hutter H. The Effect of Bias-Temperature Stress on Na+ Incorporation into Thin Insulating Films. Anal. Bioanal. Chem. 2011, 400 (3), 649–657. 10.1007/s00216-011-4686-z. 21331494

[ref78] Utke I.; Hoffmann P.; Melngailis J. Gas-Assisted Focused Electron Beam and Ion Beam Processing and Fabrication. J. Vac. Sci. Technol., B:Microelectron. Nanometer Struct.--Process., Meas., Phenom. 2008, 26 (4), 1197–1276. 10.1116/1.2955728.

