# Supplementary material for: Operando Scanning Electron Microscopy Study of Support Interactions and Mechanisms of Salt-Assisted WS2 Growth
Source: Chem Mater. 2025 Jan 30;37(3):989–1000. doi: 10.1021/acs.chemmater.4c02603 (PMC11823408; doi:10.1021/acs.chemmater.4c02603)
Supplement: Supplementary file 1 — cm4c02603_si_001.pdf [file cm4c02603_si_001.pdf]

Supporting Information

Operando Scanning Electron Microscopy Study of Support Interactions and Mechanisms of Salt-Assisted WS_2 Growth

Jinfeng Yang,[1] Ye Fan,[1] Ryo Mizuta,[1] Max Rimmer,[2] Jack Donoghue,[2] Shaoliang Guan,[3] Sarah J. Haigh,[2] Stephan Hofmann[1]

[1] Department of Engineering, University of Cambridge, Cambridge CB3 0FA, UK

[2] Department of Materials, University of Manchester, Manchester M13 9PL, UK

[3] Maxwell Centre, Cavendish Laboratory, University of Cambridge, Cambridge CB3 0HE, UK

SI Video 1: The melting process of a Na_2WO_4 particle on sapphire, recorded by operando SEM using the In-Lens SE detector (Zeiss Gemini 300 SEM, 5 kV beam voltage, 30 µm aperture size, 13 mm working distance; the sample was inside a Kammrath & Weiss 1050 Heating Module, same as for videos below) with continuous scanning at 0.7 s/frame scan rate (the video is ~18 times of the real speed). Corresponding to Fig. 2a.

SI Video 2: Example 1 of the WS_2 growth process (Stage 2), at 700 °C under 0.1 mbar DMDS (nozzle height, H = 500 µm), recorded by operando SEM using the In-Lens SE detector with continuous scanning at 0.7 s/frame scan rate. Corresponding to Fig. 3a.

SI Video 3: Example 2 of the WS_2 growth process (Stage 2), at 700 °C under 0.1 mbar DMDS (nozzle height, H = 500 µm), recorded by operando SEM using the In-Lens SE detector with continuous scanning at 0.7 s/frame scan rate. Corresponding to Fig. S9, Example 2.

SI Video 4: Example 3 of the WS_2 growth process (Stage 2), at 700 °C under 0.1 mbar DMDS (nozzle height, H = 500 µm), recorded by operando SEM using the In-Lens SE detector with continuous scanning at 0.7 s/frame scan rate. Corresponding to Fig. S9, Example 3.

SI Video 5: Example 4 of the WS_2 growth process (Stage 2), at 700 °C under 0.02 mbar DMDS (nozzle height, H = 1000 µm), recorded by operando SEM using the In-Lens SE detector with continuous scanning at 0.7 s/frame scan rate. Corresponding to Fig. S9, Example 4.

SI Video 6: The melting process of Na_2WO_4 particles in the vicinity of a scratched trench on sapphire, recorded by operando SEM using the In-Lens SE detector with continuous scanning at 0.7 s/frame scan rate (the video is ~25 times of the real speed).

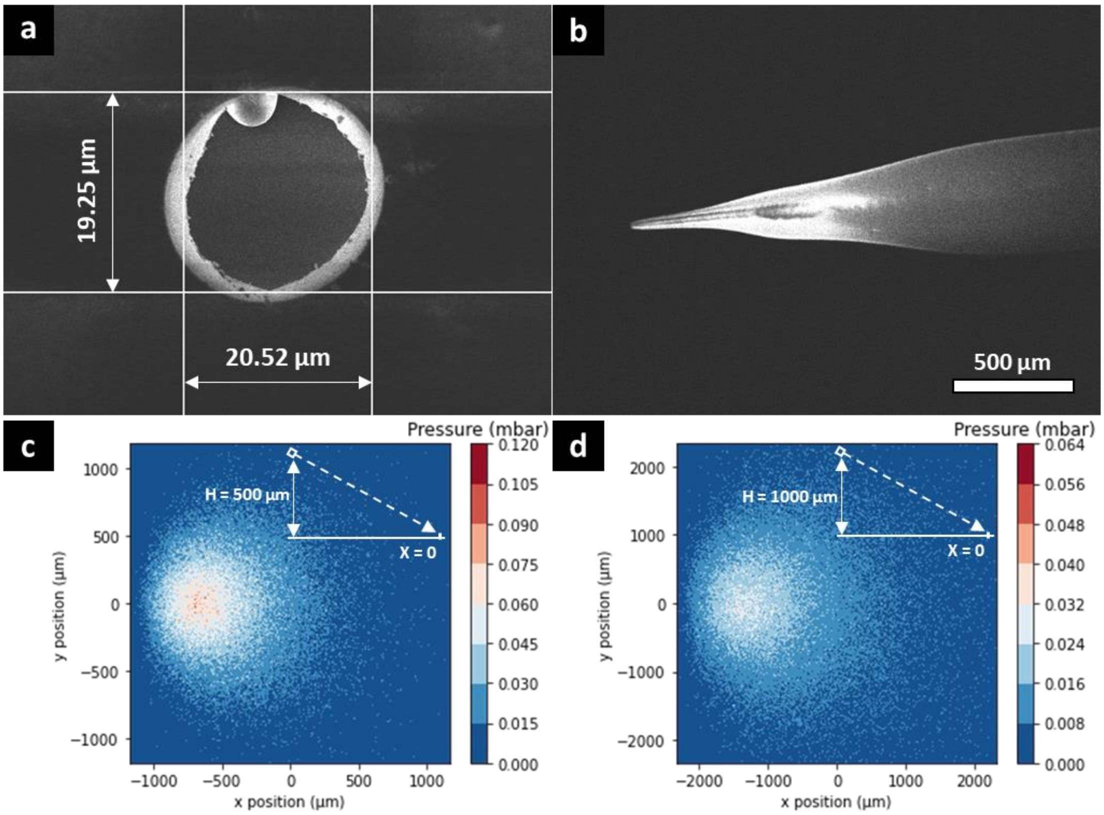

Figure S1 (a,b) SE images of the (a) top-view and (b) side-view of the µ-l nozzle. (c,d) TPMC simulation of the DMDS pressure distribution on the samples with the nozzle (c) 500 µm and (d) 1000 µm away from the sample. The insets in (c) and (d) schematically show a sideview of the relative position of the nozzle exit to the x = 0 point on the substrate.

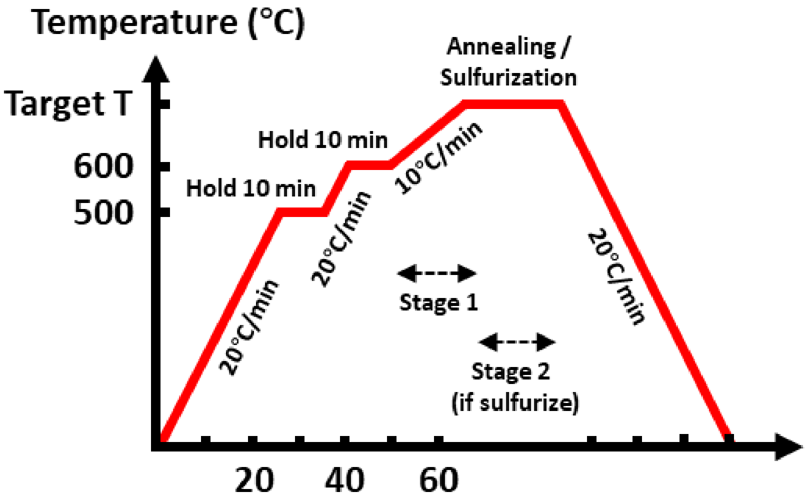

Figure S2 Typical heating profile for the operando SEM processing.

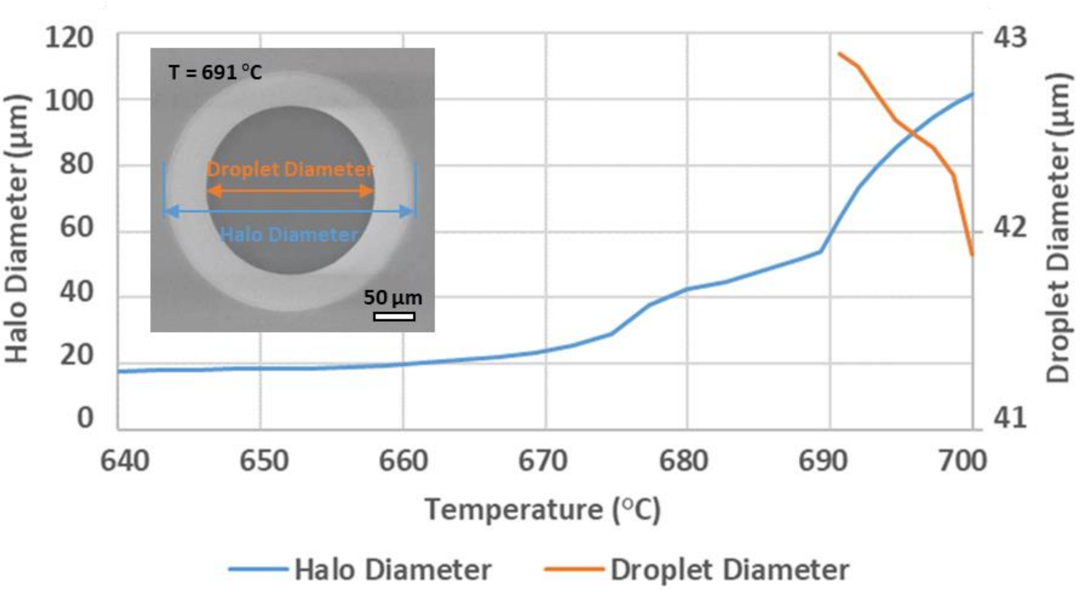

Figure S3 Diameters of the halo and droplet in Fig. 2a and SI Video 1 as a function of temperature during the melting process (Stage 1).

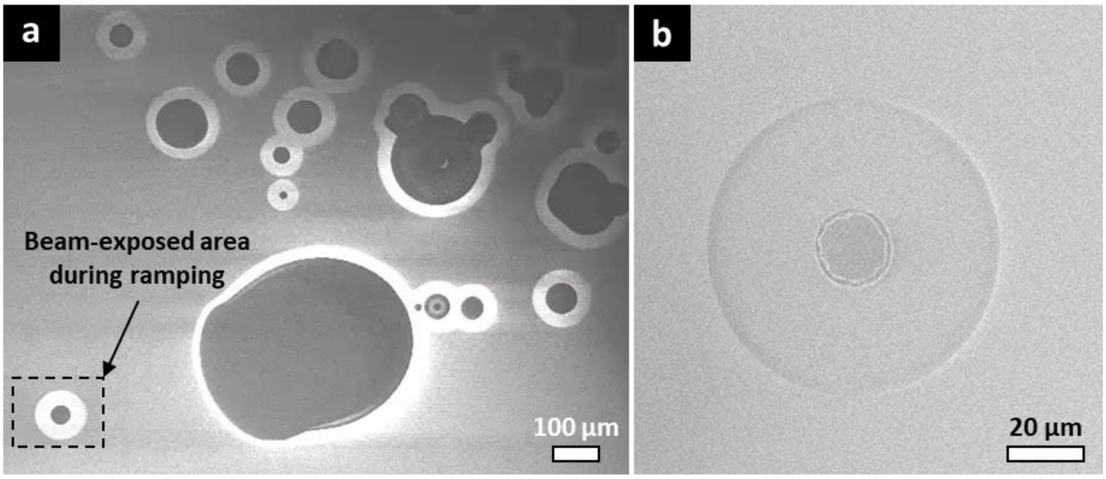

Figure S4 (a) Low magnification SE image at 700 °C showing a small area (highlighted; corresponding to Fig. 2a) that was continuously exposed by SEM compared to wider area with no prior beam exposure. (b) Room temperature SE image showing a resolidified salt particle and its halo on a sapphire sample annealed in a cold-wall CVD reactor for comparison (see Experimental Section).

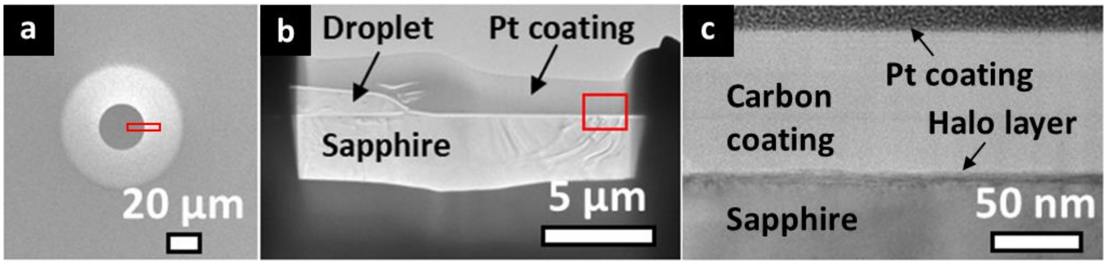

Figure S5 (a) Top-view SE image of the halo (T = 700 °C) for the cross-sectional TEM corresponding to Figure 2c. The red box marks the area for focused ion beam (FIB) cutting; (b) Cross-sectional TEM of the whole sample after FIB cutting; (c) TEM image of the area marked by the red box in (b) and corresponding to the area in Figure 2c.

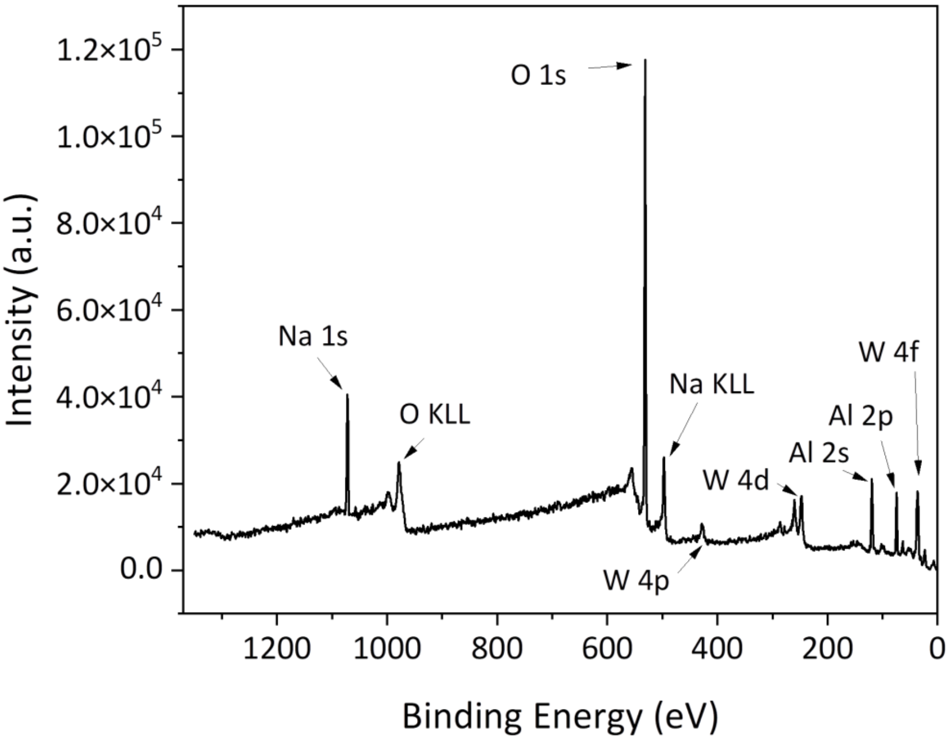

Figure S6 XPS survey taken at Point 3 as illustrated in the inset in Figure 2d.

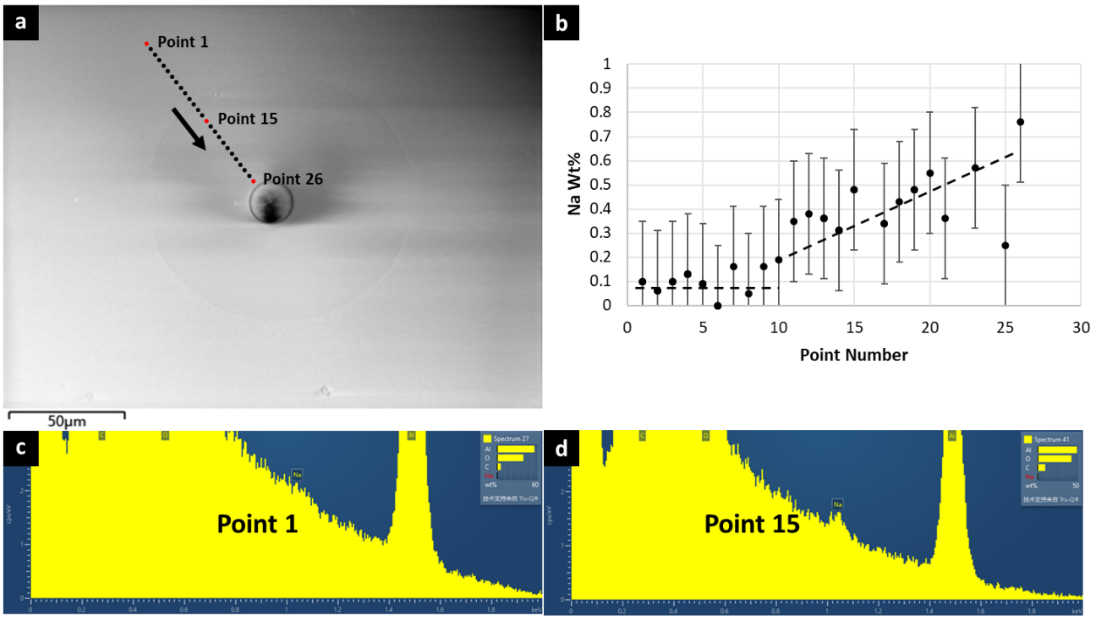

Figure S7 EDS point measurements on the surface of a halo. (a) SE image of the halo (room temperature, 1.5 kV beam voltage, ZEISS Merlin SEM); (b) Na Wt% of Point 1 to Point 26 marked in (a); (c,d) zoomed-in spectra of Point 1 and Point 15 showing the Na Kα position.

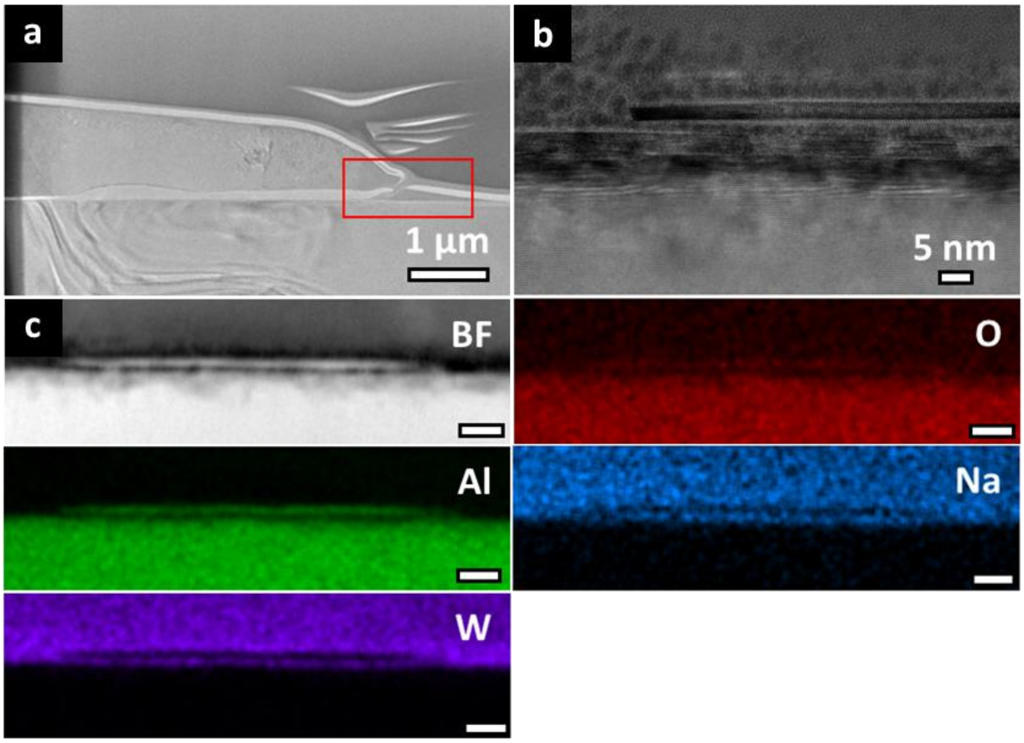

Figure S8 (a) TEM image of the sample shown in SI Fig. S5b; (b) higher magnification TEM image of the area marked by the red box in (a); (c) bright field (BF) STEM and STEM-mode EDS maps of O, Al, Na, and W of the area with a lifted-off Al_2O_3 crystal. Scale bar = 20 nm.

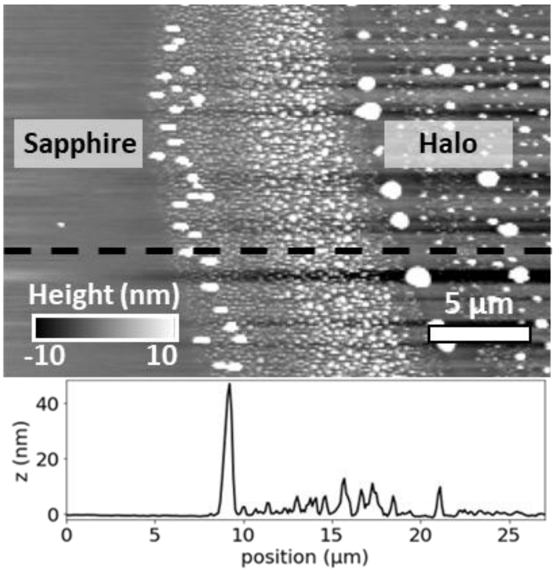

Figure S9 Post-annealing AFM map across the rim of a halo of a sodium tungstate droplet supported on sapphire, annealed at 700 °C for 40 minutes in the SEM and exposed in air for 3 weeks after cooling. The dashed line in the map indicates location of the shown line scan.

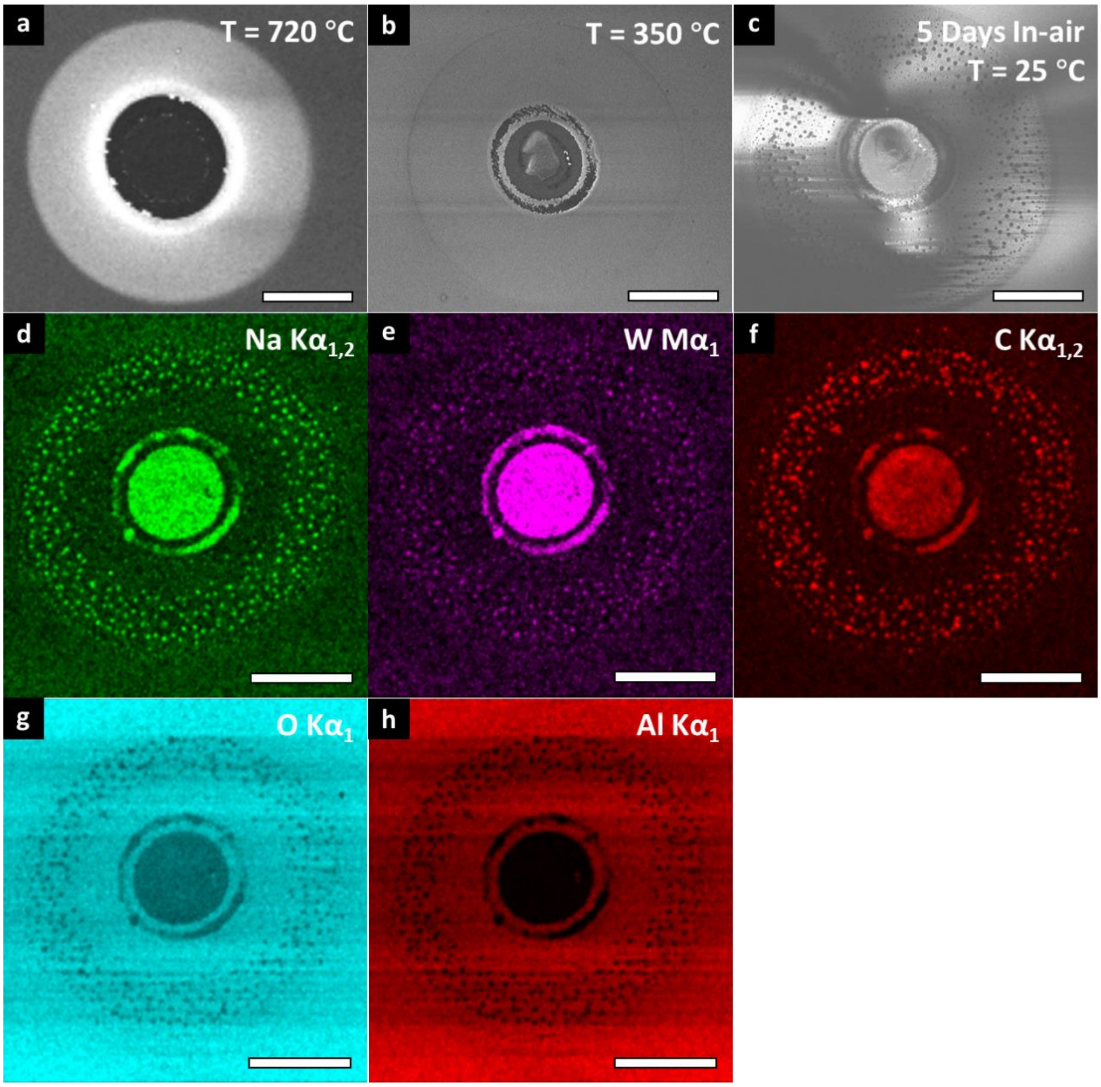

Figure S10 (a-c) SE images of a Na_2WO_4 particle (annealed at 720 °C for 10 minutes then cooled down) (a) at 720 °C after 10 minutes annealing, (b) cooled down to 350 °C, and (c) exposed in air for 5 days and at room temperature; (d-h) post-annealing room temperature EDS maps of (d) Na $K\alpha_{1,2}$, (e) W $M\alpha_1$, (f) C $K\alpha_{1,2}$, (g) O $K\alpha_1$, and (h) Al $K\alpha_1$ of the salt particle. Scale bars = 50 μm.

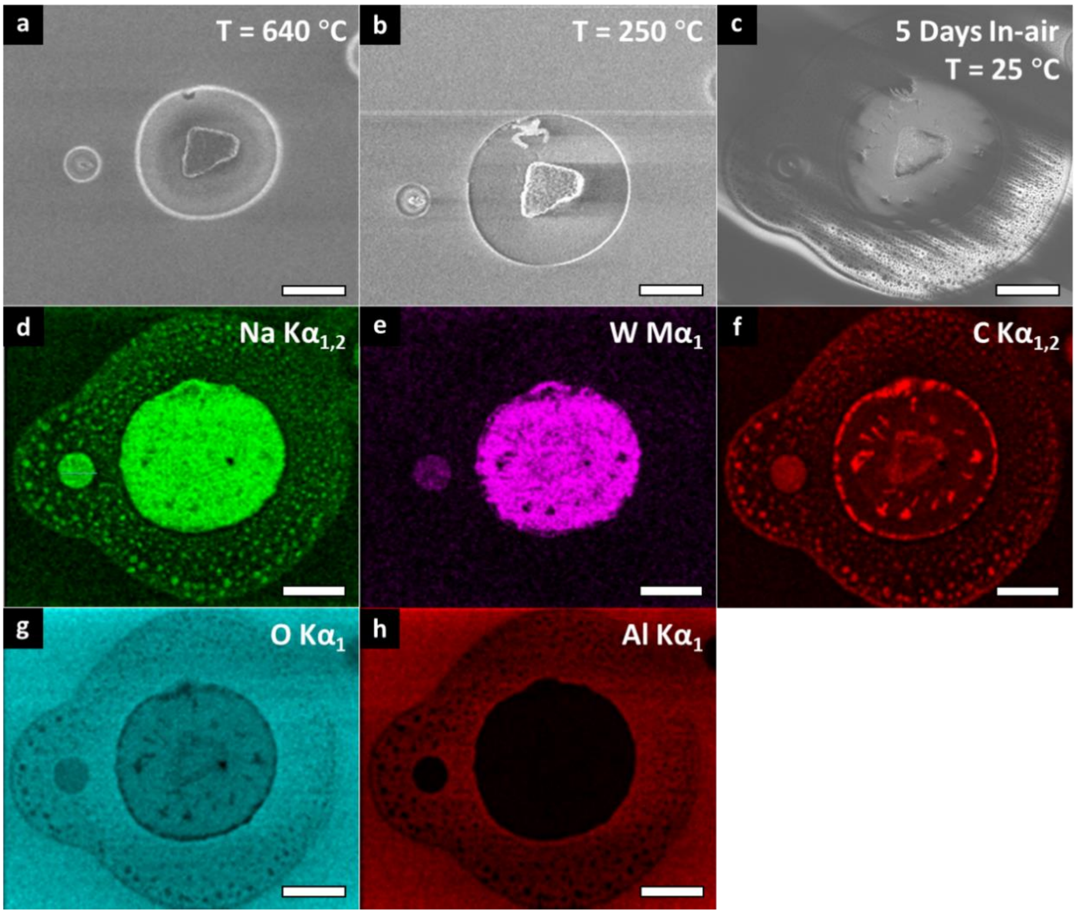

Figure S11 (a-c) SE images of a partly molten Na_2WO_4 particle on sapphire at (a) 640 °C, (b) cooled down to 250 °C, and (c) exposed in air for 5 days at room temperature; (d-h) EDX maps of (d) Na K$\alpha_{1,2}$, (e) W Mα_1, (f) C K$\alpha_{1,2}$, (g) O Kα_1, and (h) Al Kα_1 of the droplet. Scale bars = 50 µm.

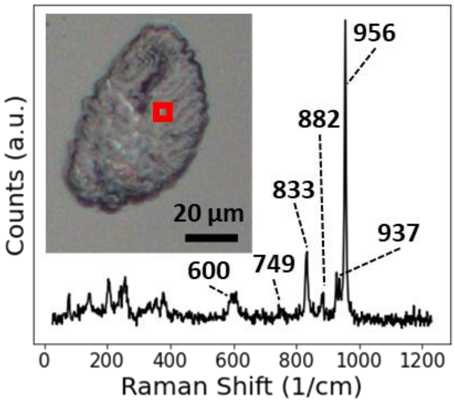

Figure S12 Post-annealing Raman spectrum of a sodium tungstate particle annealed at 700 °C on sapphire in the SEM for 3 hours. Inset figure: optical image of the particle, with the red box highlighting the Raman measurement point.

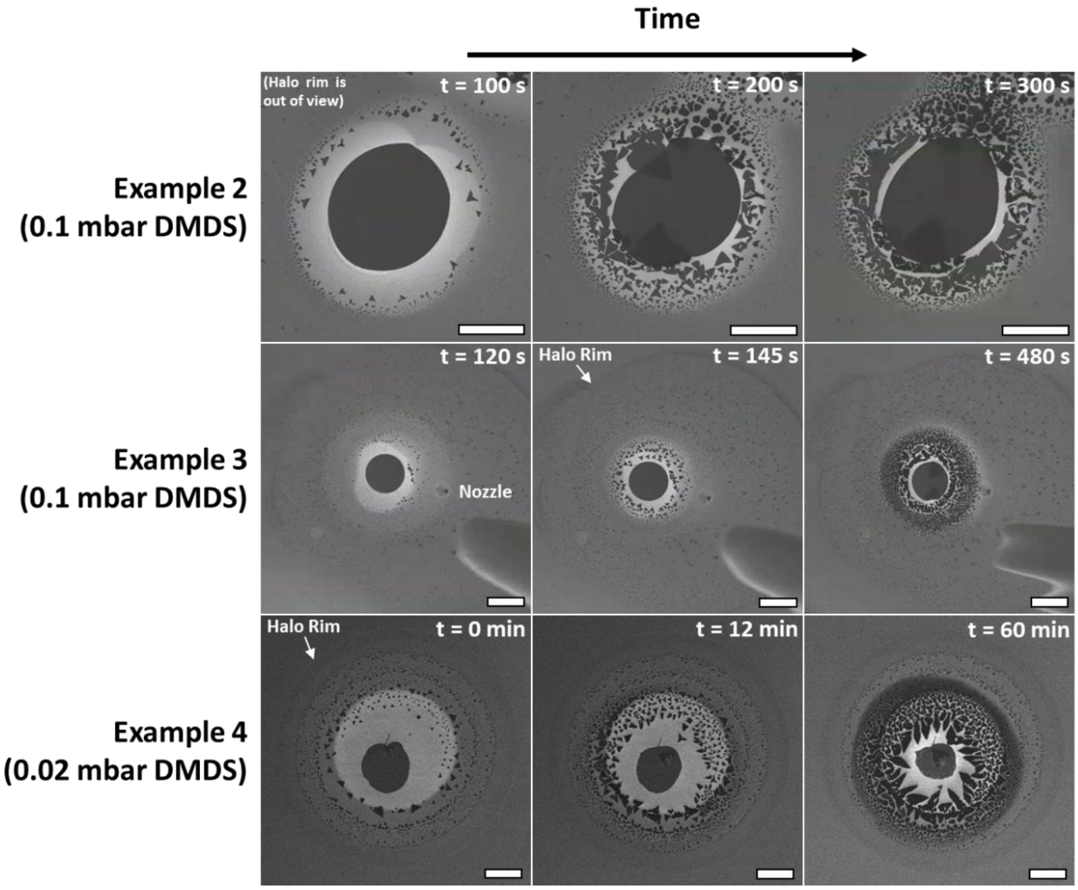

Figure S13 SE image sequences of WS_2 nucleation and growth (Stage 2). Growth runs in Examples 2 and 3 were under 0.1 mbar DMDS at 700 °C, corresponding to SI Videos 3 and 4. Growth in Example 4 was under 0.02 mbar DMDS at 700 °C, corresponding to SI Video 5. All Scale bars = 20 µm.

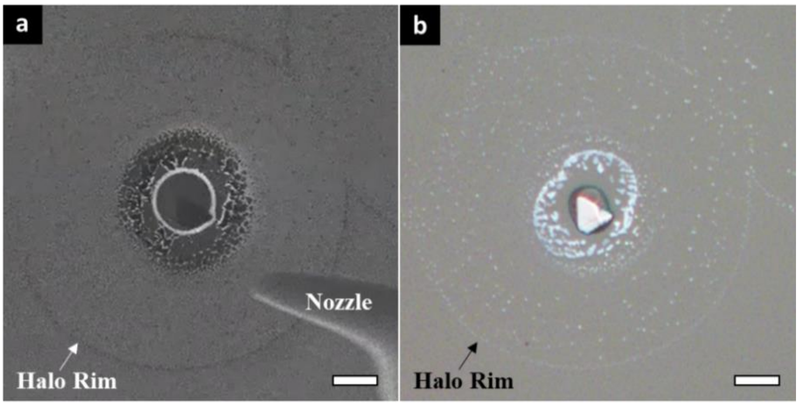

Figure S14 (a) Post-growth low magnification SE image of the growth region in Fig. 3a, at 700 °C; (b) Optical image of the region in (a) after 1 day of air exposure. Scale bars = 20 µm.

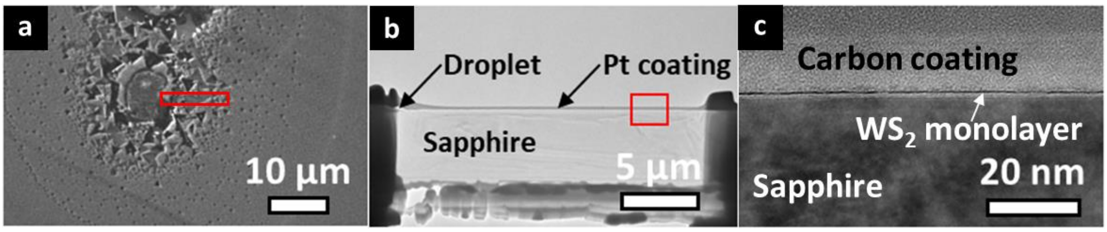

Figure S15 (a) Top-view SE image of WS_2 grown on sapphire in the halo area, corresponding to the TEM and STEM-mode EDS area. The red box marks the area for the FIB cutting; (b) Cross-sectional TEM of the whole sample after the FIB cutting; (c) TEM image of the area marked by the red box in (b).

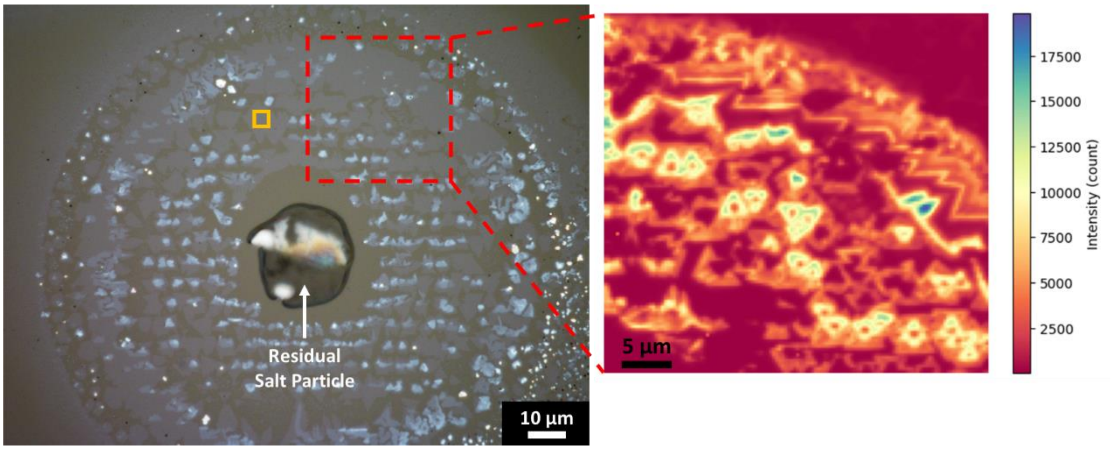

Figure S16 Wider-view optical image of the as-grown WS_2 (after 2 days of air exposure) shown in Fig. 3c (Left) with PL peak intensity map of the area marked by the red box (Right). The area marked by the yellow box corresponds to the area for the Raman and PL in Figure 3d.

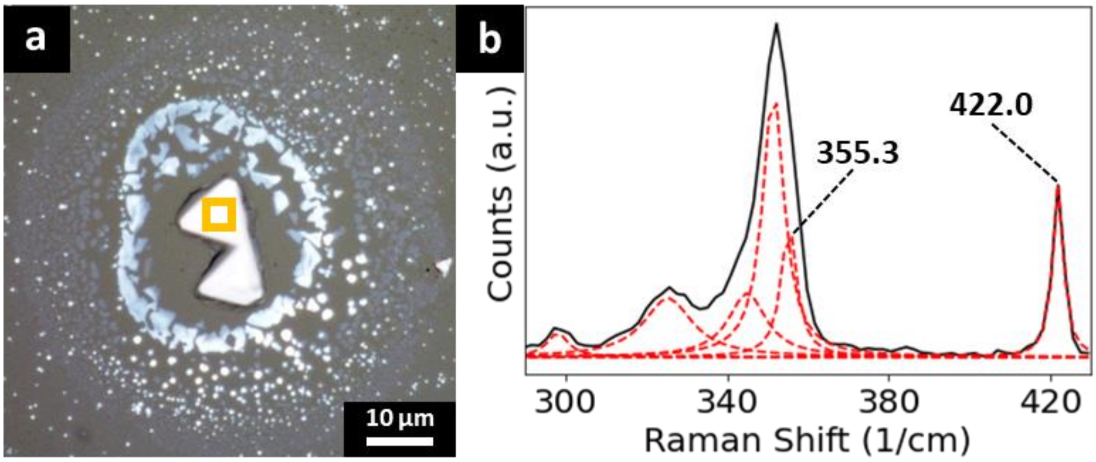

Figure S17 (a) Optical image of WS_2 grown at 700 °C under 0.1 mbar DMDS exposure on sapphire in the SEM. The image was taken after 1 day of air exposure. (b) Raman spectrum of the area marked by the yellow box in (a).

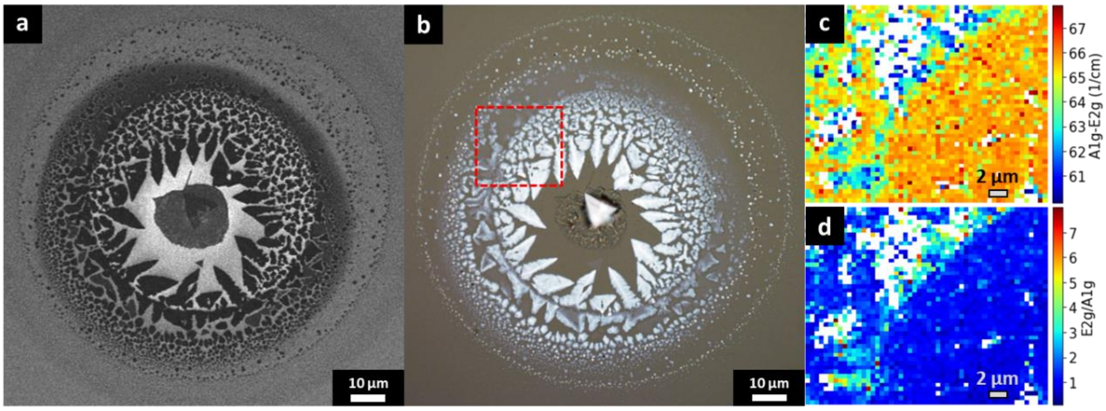

Figure S18 (a) Operando SE image of the as-grown WS_2 (after 1 hour 0.02 mbar DMDS exposure) shown in SI Figure S9 Example 4, taken at 700 °C. (b) Room temperature optical image of the as-grown WS_2, taken after 1 day of air exposure. Raman mapping of (c) $A_{1g}(\Gamma)$-$E^1_{2g}(\Gamma)$ peak distance and (d) $E^1_{2g}(\Gamma)/A_{1g}(\Gamma)$ peak ratio of the area in (b) marked by the red square. The white pixels represent the points of no WS_2 signal or where the signal is too weak to fit.

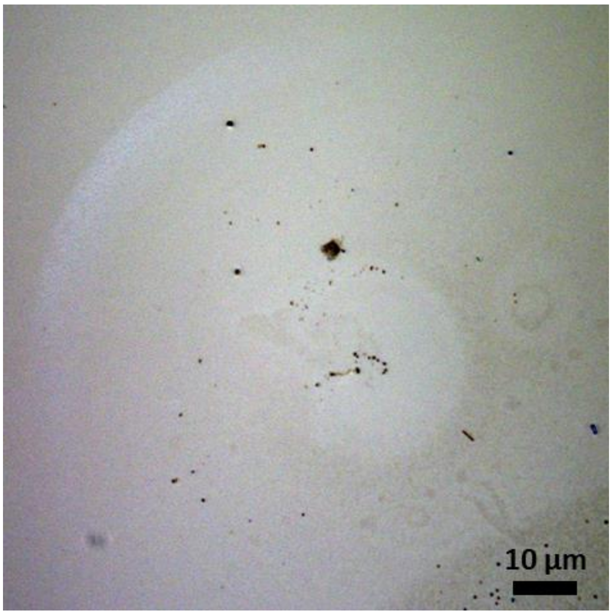

Figure S19 Optical image of the as-grown WS_2 on the 'wash-off' sample, corresponding to the SE image shown in Figure 4 (b).

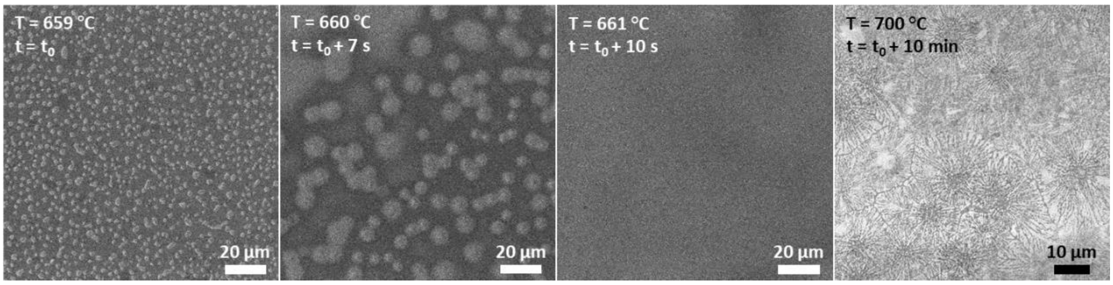

Figure S20 SE image sequence of spin-coated Na_2WO_4 melting on SiO_2/Si support during ramping and under 0.01 mbar DMDS. Recorded using 60 µm aperture and high-current mode (Zeiss Gemini 300 SEM).

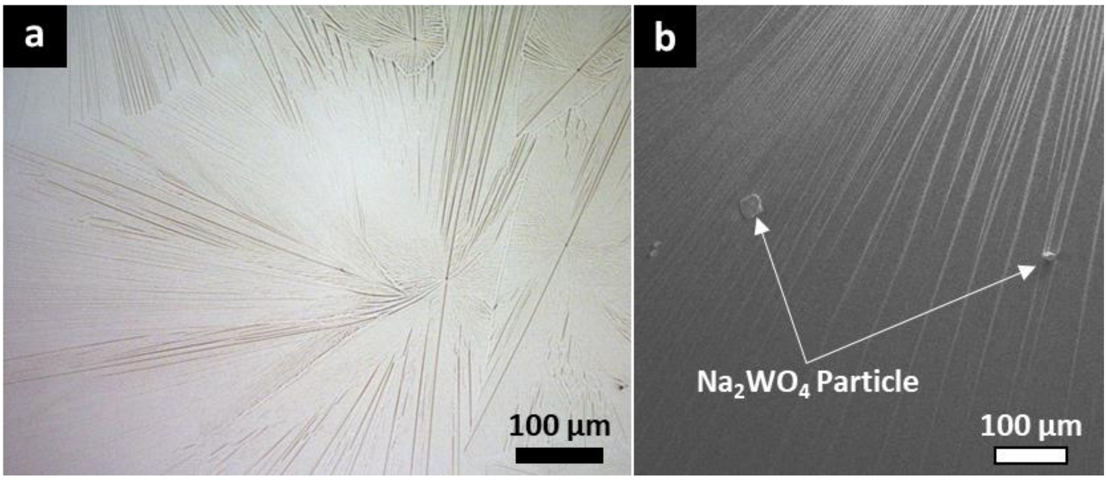

Figure S21 (a) Optical image of the NaOH-treated sapphire. (b) SE image of the NaOH-treated sapphire with Na_2WO_4 particles at 150 °C, corresponding to the area in Fig. 5c.

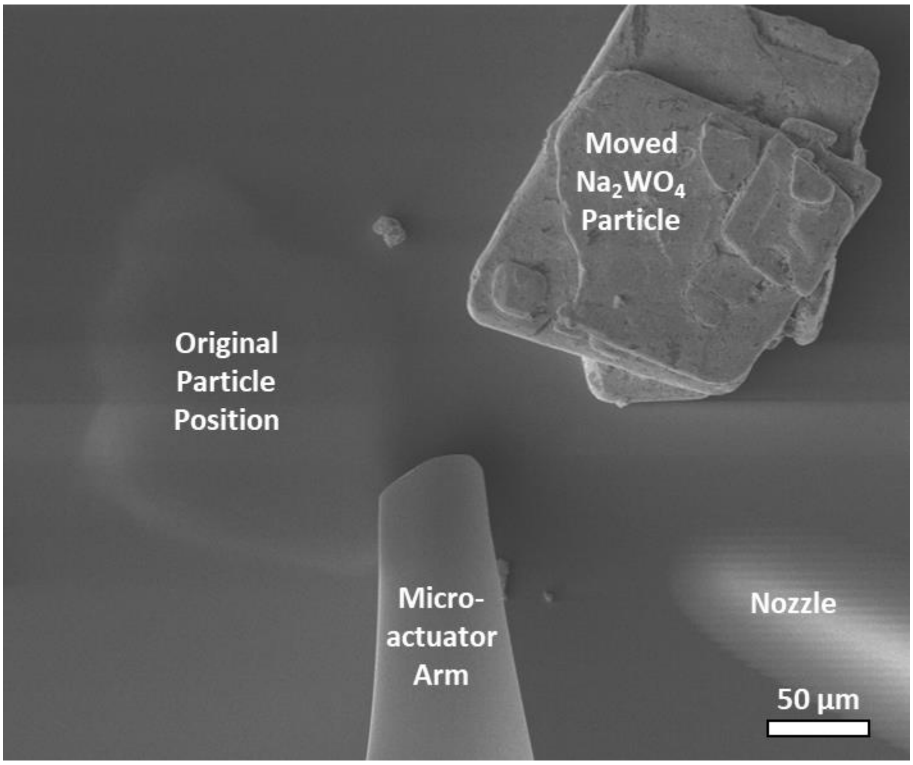

Figure S22 SE image of a Na_2WO_4 particle moved away from its original position (labelled in the figure) by the quartz micro-actuator arm (labelled in the figure). The image was taken at 500 °C, and the particle was annealed at 500 °C at its original position for 10 minutes before being moved.
